# The adverse events of CDK4/6 inhibitors for HR+/ HER2- breast cancer: an umbrella review of meta-analyses of randomized controlled trials

**DOI:** 10.3389/fphar.2024.1269922

**Published:** 2024-01-15

**Authors:** Dongqing Pu, Yue Wu, Debo Xu, Guangxi Shi, Hanhan Chen, Dandan Feng, Mengdi Zhang, Jingwei Li

**Affiliations:** ^1^ Department of First Clinical Medical College, Shandong University of Traditional Chinese Medicine, Jinan, Shandong, China; ^2^ Department of College of Traditional Chinese Medicine, Shandong University of Traditional Chinese Medicine, Jinan, Shandong, China; ^3^ Department of Breast Thyroid Surgery, Affiliated Hospital of Shandong University of Traditional Chinese Medicine, Jinan, Shandong, China

**Keywords:** CDK4/6 inhibitor, adverse event, umbrella review, randomized controlled trial, endocrine therapy, HR+/ HER2-breast cancer

## Abstract

**Background:** The clinical selection of three CDK4/6 inhibitors presents a challenging issue, owing to the absence of distinct clinical case characteristics, biomarkers, and their comparable clinical benefits in progression-free survival and overall survival To inform clinical treatment decisions, we conducted a comprehensive evaluation of the adverse events associated with CDK4/6 inhibitors in combination with endocrine therapy for hazard ratio+/HER2-breast cancer.

**Methods:** We searched Cochrane, PubMed, Embase, and Web of Science databases from their inception until 1 August 2022. The results were summarized narratively, and we assessed the methodological quality, reporting quality, and evidence quality of AEs by AMSTAR-2, PRISMA, and GRADE.

**Results:** Our analysis included 24 meta-analyses systematic reviews that evaluated the quality of AEs in 13 cases of early breast cancer (EBC) and 158 cases of advanced breast cancer The addition of CDK4/6 inhibitors was found to significantly increase AEs of any grade and AEs of grade 3 or higher in early breast cancer, along with a significant increase in the risk of treatment discontinuation. In advanced breast cancer, high and moderate-quality evidence indicated that CDK4/6 inhibitors significantly increased AEs across all grades, including grade 3/4 AEs, leucopenia, grade 3/4 leucopenia, neutropenia, grade 3/4 neutropenia, anemia, grade 3/4 anemia, nausea, grade 3/4 constipation, fatigue, pyrexia, venous thromboembolism abdominal pain, and cough. However, they did not significantly elevate the incidence of grade 3/4 diarrhea. Subgroup analysis revealed that palbociclib primarily increased hematologic toxicity, particularly grade 3/4 neutropenia, anemia, and thrombocytopenia. Ribociclib was mainly associated with grade 3/4 neutropenia, prolonged QT interval, and alopecia. Abemaciclib was closely linked with diarrhea and elevated blood creatinine levels.

**Conclusion:** The AEs associated with CDK4/6 inhibitors vary, necessitating individualized and precise clinical selection for optimal management. This approach should be based on the patient’s medical history and the distinct characteristics of different CDK4/6 inhibitors to improve the patient’s quality of life.

**Systematic Review Registration**: [https://systematicreview.gov/], identifier [CRD42022350167]

## 1 Introduction

One of the primary factors contributing to the recurrence and metastasis of hormone receptor-positive (HR+)/HER2-negative (HER2-) early breast cancer (EBC) and advanced breast cancer (ABC) is resistance to endocrine therapy (ET) (Jeselsohn et al., 2015). Due to significant advancements in molecular biology, molecular targeted therapy for breast cancer has become increasingly popular. Notably, CDK4/6 inhibitors represent a major breakthrough in overcoming ET resistance and reducing the recurrence and metastasis of breast cancer. Numerous global, multicenter, clinical randomized controlled trials (RCTs) (PALOMA, MONARCH, MONALEESA, PALLAS, PENELOPE-B, etc.) conducted from 2014 to the present have investigated the efficacy of CDK4/6 inhibitors in combination with ET for HR+/HER2- EBC and ABC(Romero, 2017; Slamon et al., 2018; Tripathy et al., 2018; Hortobagyi et al., 2019; Loibl et al., 2021; Mayer et al., 2021). These combination therapies have significantly improved clinical prognosis. Consequently the Food and Drug Administration (FDA) approved three CDK4/6 inhibitors, palbociclib, ribociclib, and abemaciclib, for use as first and second-line treatments for HR+/HER2-metastatic breast cancer (MBC), based on these promising clinical trials (Mullard, 2017; Burstein et al., 2021). Additionally, abemaciclib received FDA approval for concurrent use with ET adjuvant treatment in patients with EBC who are HR+/HER2-, Ki-67 ≥ 20%, lymph node positivity, and at high risk of recurrence (Royce et al., 2022).

The safety profile of CDK4/6 inhibitors has garnered considerable attention. A thorough assessment of the adverse events (AEs) associated with CDK4/6 inhibitors can enhance clinical decision-making, monitoring, and management, thereby improving patient compliance and quality of life. Numerous systematic reviews/meta-analyses (SRs/MAs) have evaluated the safety of CDK4/6 inhibitors in combination with ET, focusing on diverse aspects such as bone marrow, gastrointestinal, skin AEs, and deep vein thrombosis (DVT) (Shohdy et al., 2017; Kassem et al., 2018; Thein et al., 2020; Silvestri et al., 2021). Nevertheless, limitations in study design, methodology, and procedures have resulted in varied evidence strengths, offering limited guidance for clinical practice. Based on this, this study is the first to summarize the AEs of CDK4/6 inhibitors combined with ET from SRs/MAs included in RCTs and to provide a comprehensive assessment using methodological quality, report quality and quality of evidence, with the aim of providing a basis for selection and reliable evidence for the clinical use of CDK4/6 inhibitors.

## 2 Materials and methods

An umbrella review of the AEs of CDK4/6 inhibitors in the treatment of breast cancer patients was conducted according to the Joanna Briggs Institute (JBI) Manual for Evidence Synthesis. The protocol has been previously registered and published in The International Prospective Register of Systematic Reviews (PROSPERO) database (CRD42022350167).

### 2.1 Search strategy

A comprehensive literature search was conducted in the Cochrane, PubMed, Embase, and Web of Science databases from their inception until August 1st. The objective was to gather literature pertaining to the use of CDK4/6 inhibitors in combination with ET for breast cancer. Both subject terms and free terms were employed in each database. Search phrases included “breast cancer," “Cyclin-Dependent Kinases 4 and 6 Inhibitors,” “Systematic review,” “Meta-analysis,” and the searches were limited to English (Supplementary Data S1).

### 2.2 Eligibility criteria

Inclusion criteria were based on the PICOS (participant, intervention, comparison, outcome, and study type) framework (P) Participants were breast cancer patients of any race, age, or disease stage (I/C) the trial group received CDK4/6 inhibitors combined with ET, while the control group received ET alone or with placebo (O) outcomes measured included AEs, with data such as risk difference (RD), relative risk (RR), odds ratio (OR), hazard ratio (HR) (S) Study types were meta-analyses (MAs) and systematic reviews (SRs) comprising exclusively RCTs.

Exclusion criteria were: (a) duplicate publications; (b) articles that did not report the necessary data; (c) systematic review reevaluation plans, conference abstracts, *etc.*

### 2.3 Literature screening and data extraction

Two reviewers (WY and XDB) independently conducted the screening and data extraction process. Initially, duplicate titles were removed, followed by screening of titles and abstracts, and then full-text evaluation based on the inclusion and exclusion criteria. Data extracted included first author, publication year, country, study population, sample size, interventions/control measures, outcome measures, and quality assessment methods. Any disagreements were resolved through the consensus of a third reviewer (CHH).

### 2.4 Data analysis

Following the Joanna Briggs Institute guidelines for Umbrella Reviews, we conducted a descriptive analysis of AEs for CDK4/6 inhibitors in combination with ET for breast cancer, without reanalyzing data from RCTs or MAs/SRs (Aromataris et al., 2015). We summarized indicators for CDK4/6 inhibitors, including RD, RR, OR, HR, 95% confidence interval (95% CI), and *p*-value. I^2^ was utilized to assess study heterogeneity, with statistical significance set at a *p*-value <0.05.

Two independent evaluators (WY and XDB) performed assessments using the Assessment of Multiple Systematic Reviews 2 (AMSTAR-2) scale, PRISMA statement, and the Grades of Recommendation, Assessment, Development, and Evaluation (GRADE) instrument and resolved disagreements by consensus of a third-party reviewer (Supplementary Data S2). First, the AMSTAR-2 scale, a systematic evaluative methodological quality assessment tool, was used to assess the study’s methodological quality (Shea et al., 2009; Shea et al., 2017). The AMSTAR-2 scale, a tool for assessing methodological quality, includes 16 items scored as “yes,” “no,” or “partially yes,” based on the criteria fulfillment. The PRISMA 2020 checklist was employed for assessing reporting quality, comprising 27 items (42 sub-item levels) (Hutton et al., 2015). Each item was scored as 1 for complete reporting, 0.5 for partial, and 0 for non-reporting, with a total possible score of 42. Scores of 33–42 indicated relatively complete reporting, 25–32 indicated some deficiencies, and scores below 25 signified serious information deficiencies (Page et al., 2021). The GRADE tool evaluated evidence quality for each outcome by examining risk of bias, inconsistency, indirectness, precision, and publication bias, categorizing outcome indicators as high, moderate, low, or very low based on downgrades (Schunemann et al., 2020; Zeng et al., 2021).

## 3 Results

### 3.1 Basic characteristics of the included literature

**FIGURE 1:**
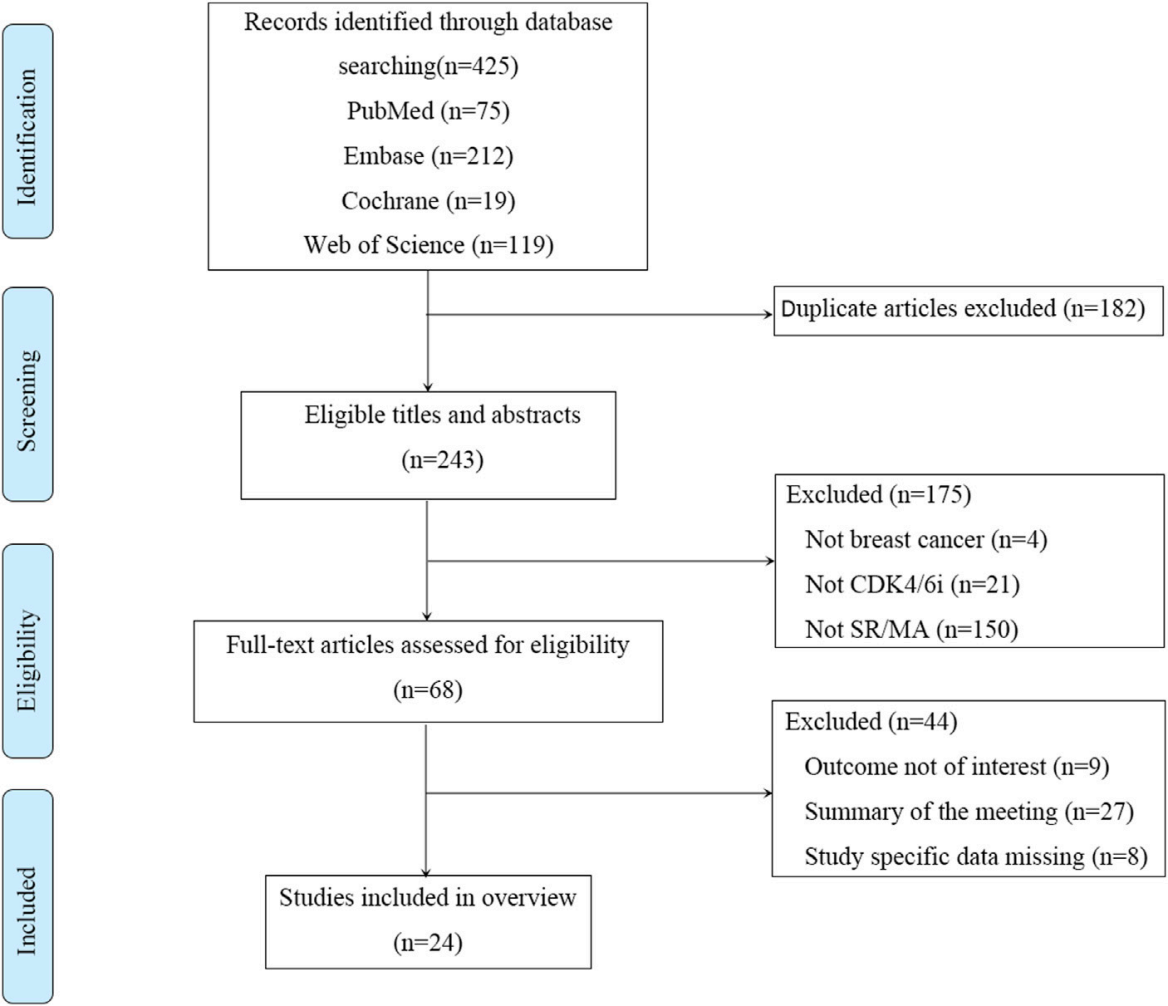
The flowchart of the literature screening. SR: systematic review; MA: meta-analysis.

From four database searches, 425 pieces of literature were initially retrieved. After removing duplicates, screening titles and abstracts, and evaluating full texts, 24 studies met the inclusion criteria. The literature screening process and results are depicted (Figure 1). Publications spanned from 2017 to 2021, with study numbers ranging from 3 to 9 and sample sizes from 1,352 to 12,647. Regarding the included population, two studies focused on HR+/HER2- EBC(Agostinetto et al., 2021; Gao et al., 2021), with the remainder addressing HR+/HER2- ABC. In terms of therapeutic interventions, one study (Ramos-Esquivel et al., 2020) compared CDK4/6 inhibitor combined with fulvestrant *versus* fulvestrant alone, two studies (Ramos-Esquivel et al., 2018; Shimoi et al., 2020) compared CDK4/6 inhibitor with AI *versus* AI alone, and the rest involved CDK4/6 inhibitor combined with ET *versus* ET treatment. For study outcomes, 11 studies reported pooled AE outcomes, such as all-grade AEs, grade 3/4 AEs, and grades 3–5 AEs (Deng et al., 2018; Martel et al., 2018; Messina et al., 2018; Ramos-Esquivel et al., 2018; Ramos-Esquivel et al., 2020; Shimoi et al., 2020; Zheng et al., 2020; Agostinetto et al., 2021; Gao et al., 2021; Li et al., 2021; Tian et al., 2021). The other studies reported specific AE outcomes without pooled AE data (Table 1).

**TABLE 1:** Main characteristics of systematic reviews and meta-analyses included in the umbrella review.

Author(s),year	Country	Trials (subjects)	Population	Experimental intervention	Control intervention	Adverse events	Quality assessment	AMSTAR2^1^	PRISMA^2^
Agostinetto et al. (2021); Agostinetto et al. (2021)	Belgium	3 (12,647)	HR+/HER2- early breast cancer	CDK4/6is^3^+ET	ET^4^	AEs^5^ (All Grade)、AEs (Grade≥3)	Cochrane criteria	L^11^	35
Gao et al. (2021); Gao et al. (2021)	China	3 (12,647)	HR+/HER2- early breast cancer	CDK4/6is + ET	ET	G3/4^6^ AEs、Hematologic AEs、Neutropenia、Leucopenia、Anemia、Lymphopenia、Thrombocytopenia、 Fatigue、Nausea、Diarrhea、Arthralgia	Cochrane criteria	VL^12^	36
Guo et al. (2019); Guo et al. (2019)	China	3 (1,352)	cancer/HR+/HER2- advanced breast cancer	palbociclib + ET	ET	Neutropenia、Leucopenia、Thrombocytopenia、Mucositis、Anemia、Alopecia、Rash、Asthenia、Fatigue、Decreased Appetite	QUADAS-2	VL	26
Lee et al. (2019); Lee et al. (2019)	Australia	4 (2499)	HR+/HER2- advanced breast cancer	CDK4/6is + ET	ET	Neutropenia	——	VL	23.5
Li et al. (2020); Li et al. (2020)	China	8 (4580)	HR+/HER2- advanced breast cancer	CDK4/6is + ET	ET	Neutropenia、Leucopenia、Anemia、Diarrhea、Fatigue、Nausea、Arthralgia	Cochrane criteria	VL	31
Li et al., 2020; Li et al., 2020	China	9 (5043)	HR+/HER2- metastatic breast cancer	CDK4/6is + ET	ET	Neutropenia、Leucopenia、Nausea、Diarrhea、Vomiting、Fatigue	——	VL	31
Li et al. (2021); Li et al. (2021)	China	7 (4415)	HR+/HER2- advanced breast cancer	CDK4/6is + ET	ET	G3-5 AEs	Cochrane criteria	VL	24
Lin et al. (2020); Lin et al. (2020))	China	6 (3421)	HR+/HER2- metastatic breast cancer	CDK4/6is + ET	ET	Neutropenia、Nausea、Fatigue、Diarrhea、Leucopenia、Vomiting、Hot Flush、Headache、Arthralgia、Anemia	Cochrane criteria	VL	30
Messina et al. (2018); Messina et al. (2018))	Italy	8 (4578)	HR+/HER2- advanced breast cancer	CDK4/6is + ET	ET	G3/4 AEs、Neutropenia	Cochrane criteria	VL	26
Ramos-Esquivel et al. (2020); Ramos-Esquivel et al. (2020)	Costa Rica	3 (1916)	HR+/HER2- metastatic breast cancer	CDK4/6is + fulvestrant	fulvestrant	AEs (All Grade)	Cochrane criteria	VL	28
Ramos-Esquivel et al. (2018); Ramos-Esquivel et al. (2018)	Costa Rica	3 (1827)	post-menopausal HR+/HER2- metastatic breast cancer	CDK4/6is + AI	AI^7^	G3/4 AEs	Cochrane criteria	VL	29
Shimoi et al. (2020); Shimoi et al. (2020))	Japan	4 (1992)	post-menopausal HR+/HER2- metastatic breast cancer	CDK4/6is + AI	AI	AEs (Grade≥3)	Cochrane criteria	VL	26
Tian et al. (2021); Tian et al. (2021))	China	8 (4580)	HR+/HER2- advanced breast cancer	CDK4/6is + ET	ET	G3/4 AEs、Neutropenia、Leucopenia、Anemia、Diarrhea、Fatigue	Cochrane criteria	VL	30.5
Xu et al. (2020); Xu et al. (2020))	China	8 (4580)	HR+/HER2- advanced breast cancer	CDK4/6is + ET	ET	Neutropenia、Leucopenia、Anemia、Nausea、Diarrhea、Vomiting	Cochrane criteria	VL	29
Yang et al. (2021); Yang et al. (2021))	China	6 (3685)	HR+/HER2- advanced breast cancer	CDK4/6is + ET	ET	Neutropenia、Leucopenia、Thrombocytopenia、Anemia、Fatigue、Diarrhea、Vomiting、Febrile Neutropenia、Nausea、Increased ALT、Increased AST、Decreased Appetite	Cochrane criteria	L	31.5
Zheng et al. (2020); Zheng et al. (2020)	China	9 (5043)	HR+/HER2- advanced breast cancer	CDK4/6is + ET	ET	G3/4 AEs、Neutropenia、Leucopenia、Anemia	Cochrane criteria	VL	30.5
Kassem et al. (2018); Kassem et al. (2018))	Egypt	6 (3178)	HR + advanced breast cancer	CDK4/6is + ET	ET	Leucopenia、Neutropenia、Thrombocytopenia、Anemia、Febrile Neutropenia	——	VL	25.5
Lasheen et al. (2017); Lasheen et al. (2017))	Egypt	4 (2007)	HR + advanced breast cancer	CDK4/6is + ET	ET	Fatigue、Alopecia、Stomatitis	——	VL	24
Shohdy et al. (2017); Shohdy et al. (2017))	Egypt	4 (2007)	HR + advanced breast cancer	CDK4/6is + ET	ET	Nausea、Vomiting、Decreased Appetite、Diarrhea	Cochrane criteria	VL	28
Thein et al. (2020); Thein et al. (2020))	United States	8 (4557)	HR+/HER2- metastatic breast cancer	CDK4/6is + ET	ET	VTE^8^	Cochrane criteria	VL	29.5
Martel et al. , 2017 ( Martel et al. (2018))	Belgium	5 (2671)	HR + metastatic breast cancer	CDK4/6is + ET	ET	AEs (All Grade)、G3/4 AEs、Fatigue、Rash、Nausea、Diarrhea、Increased AST^9^、Increased ALT^10^、Leucopenia、Neutropenia、Febrile Neutropenia、Thrombocytopenia、Anemia	——	VL	25.5
Toss et al. (2019); Toss et al. (2019))	Italy	4 (2499)	HR + Bone-Only Metastatic Breast Cancer	CDK4/6is + ET	ET	Alopecia、Anemia、Leucopenia、Neutropenia、Nausea、Vomiting、Decreased Appetite、Fatigue、Diarrhea、Headache、Constipation、Pyrexia、Stomatitis、Thrombocytopenia、Cough、Abdominal Pain、Asthenia、Increased AST、Pain In Extremities、Arthralgia、Back Pain、Hot Flush、Increased ALT、Rash	——	VL	26
Deng et al. (2018); Deng et al. (2018))	China	7 (3854)	HR+/HER2- advanced breast cancer	CDK4/6is + ET	ET	AEs (All Grade)、G3/4 AEs、Neutropenia、Leucopenia、Anemia、Alopecia、Diarrhea、Vomiting、Nausea、Fatigue、Injections、Headache、Arthralgia、Hot Flush	Cochrane criteria	VL	26.5
Bolzacchini et al. (2021); Bolzacchini et al. (2021))	Italy	7 (4415)	HR+/HER2- advanced breast cancer	CDK4/6is + ET	ET	VTE、All Arterial Events、Myocardial、Angina、Cerebral Ischemia Infarction、Angina、	Cochrane criteria	VL	27.5

Abbreviations: AMSTAR-2, assessment of mutiple systematic reviews-2; PRISMA, preferred reporting items for systematic reviews and meta-analyses; CDK4/6is, CDK4/6 inhibitors; ET, endocrine therapy; AEs, adverse events; G3/4, grade 3/4; AI, aromatase inhibitors; VTE, venous thromboembolism; AST, aspartate aminotransferase; ALT, alanine aminotransferase; L, low; VL, very low.

### 3.2 Methodological quality of the included studies

**FIGURE 2:**
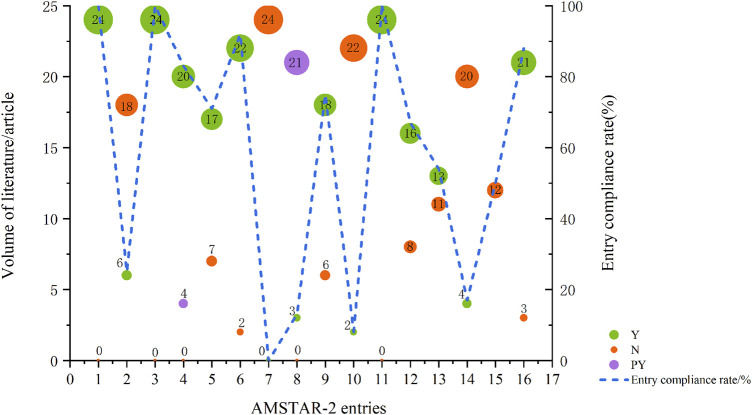
Results of the AMSTAR-2 assessment. Abbreviations: Y, Yes; N, No; PY, Partial Yes; each entry is Y for full compliance, PY for partial compliance, and N for non-compliance; entry compliance rate = (number of documents complying with this entry/total documents) * 100%.

AMSTAR-2, an extensive critical appraisal tool, facilitates rapid and reproducible assessments of systematic reviews of randomized controlled trials (RCTs) for interventions, thereby identifying high-quality evaluations for decision-makers (Shea et al., 2017). We utilized the AMSTAR-2 scale to appraise the methodological quality of the included studies. Two studies were evaluated as low quality (Agostinetto et al., 2021; Yang et al., 2021), while the remainder were deemed very low quality. For specific AMSTAR-2 criteria, entries 1, 3, and 11 achieved 100% compliance, indicating all included PICO, specified the types of literature included, and employed appropriate statistical methods for combined result analysis. Conversely, entries 7, 8, 10, and 14 had compliance rates of 0%, 13%, 8%, and 17%, respectively, highlighting significant gaps in justifying the inclusion of study types, listing and explaining excluded literature, detailing study characteristics, reporting funding sources, and elucidating result heterogeneity (Table 1; Figure 2, and Supplementary Data S3).

### 3.3 Methodological quality of the included studies

**FIGURE 3:**
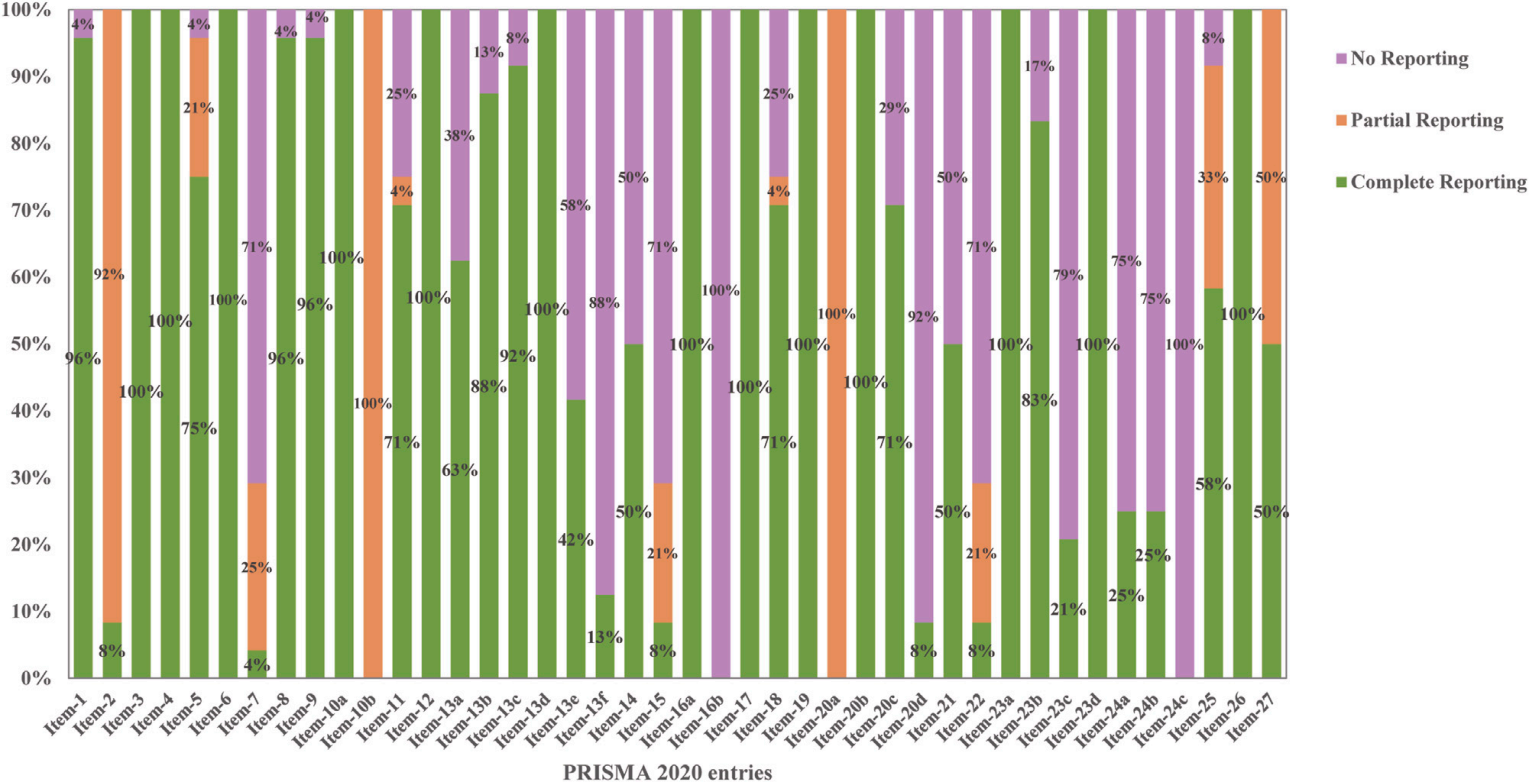
Results of the PRISMA 2020 assessment.

PRISMA 2020 serves as a reporting guideline for systematic reviews of health intervention studies, regardless of study design. Its comprehensive reporting allows readers to evaluate the methodological soundness and credibility of study results (Page et al., 2021). We applied the PRISMA checklist to assess the reporting quality of the 24 included papers, which scored between 23.5 and 36. Two reports, scoring from 33 to 42, were relatively complete (Agostinetto et al., 2021; Gao et al., 2021). Nineteen reports, scoring from 25 to 32, exhibited some deficiencies. Three reports (Lasheen et al., 2017; Lee et al., 2019; Li et al., 2021) scoring less than 25 had serious deficiencies. Common omissions included item 7 (detailing the search strategy for databases), 13f (conducting sensitivity analysis in synthesis methods), 15 (assessing certainty), 16b (explaining literature exclusion reasons in results), 20d (providing sensitivity analysis in synthesis results), 22 (providing evidence certainty in results), 23c (discussing any limitations in the review process), and 24abc (providing registration and protocol-related information) (Figure 3; Table 1 and Supplementary Data S4).

### 3.4 Adverse event

**FIGURE 4:**
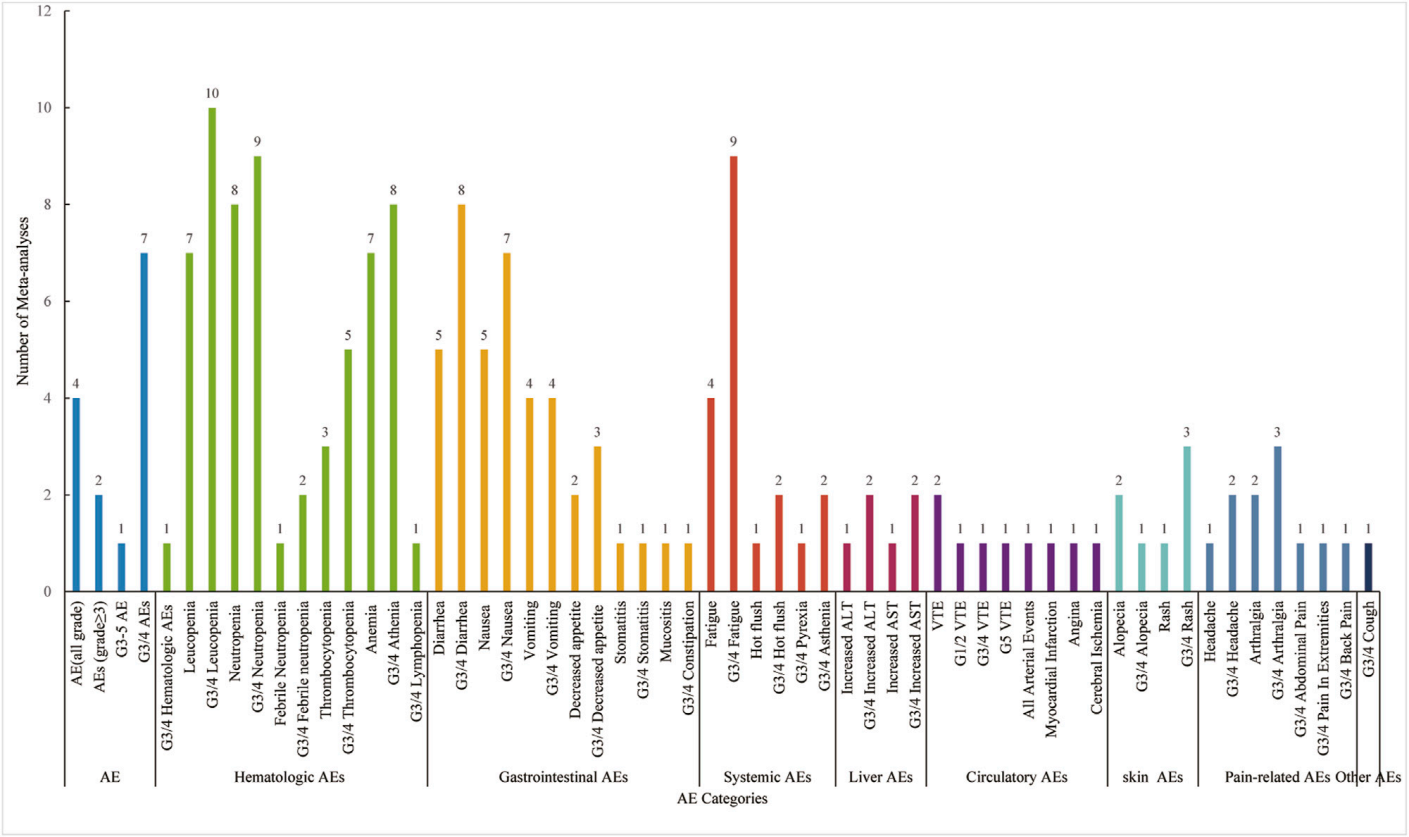
Summary of adverse events of CDK4/6 inhibitors combined with ET treatment.

Patients treated with CDK4/6 inhibitors, compared to ET alone, experienced a higher incidence of AEs. The main AE categories were distributed across nine areas (n = number of MAs): summary AEs (n = 14), hematological AEs (n = 15), gastrointestinal AEs (n = 13), systemic AEs (n = 11), liver AEs (n = 2), circulatory AEs s (n = 2), skin AEs (n = 5), pain-related AEs (n = 5) and other types AEs (n = 2). Notably, hematological and gastrointestinal AEs were more prevalent, with frequent reports of leucopenia, neutropenia, thrombocytopenia, anemia, diarrhea, nausea, and vomiting. Among systemic AEs, reports of fatigue were substantial (Figure 4).

#### 3.4.1 AEs in early breast cancer

**FIGURE 5:**
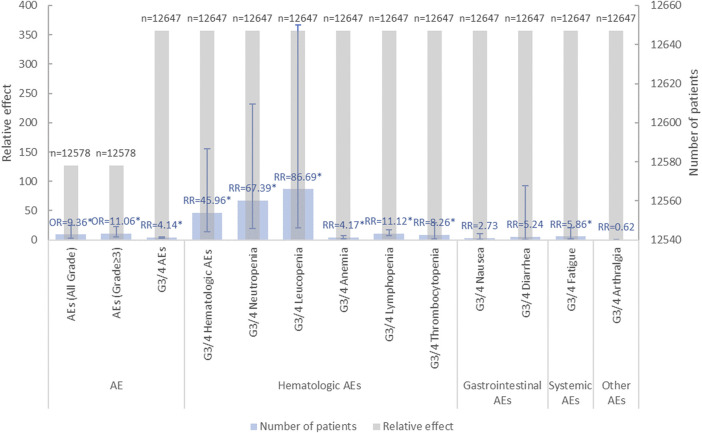
Summary chart of adverse events in EBC.

A total of 2 MAs for EBC investigated the AEs of CDK4/6 inhibitors in combination with ET, focusing on summary AEs, hematologic AEs, gastrointestinal AEs, systemic AEs, and other AEs (Agostinetto et al., 2021; Gao et al., 2021). Adjuvant CDK4/6 inhibitors were notably associated with an increased overall incidence of all-grade AEs and grade 3 AEs, although the results showed considerable heterogeneity and the evidence quality was extremely low, based on data pooled from two studies. Gao HF’s research highlighted a focus on hematologic and systemic AEs, especially grade 3/4 leucopenia, neutropenia, lymphopenia, thrombocytopenia, and fatigue. However, the incidence of other AEs did not significantly differ between combination therapy and ET alone (Gao et al., 2021). Gao HF also conducted a subgroup analysis of CDK4/6 inhibitors, finding no significant differences in grade 3/4 AEs between abemaciclib and palbociclib. Agostinetto E et al. reported that adding a CDK4/6 inhibitor to ET significantly increased the likelihood of early treatment cessation (OR = 22.11, 95%CI: 9.45–51.69, *p* < 0.001) (Agostinetto et al., 2021) (Figure 5, Supplementary Data S5).

#### 3.4.2 AEs in advance breast cancer

##### 3.4.2.1 Summary AEs

**FIGURE 6:**
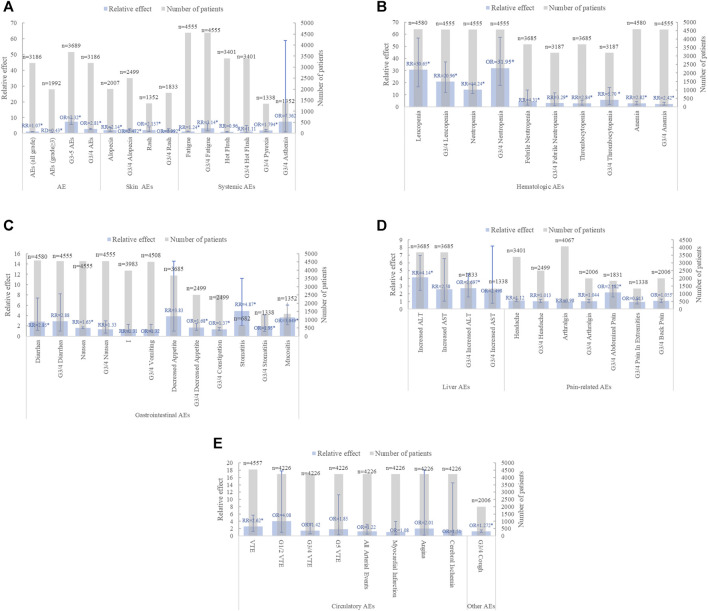
Summary chart of adverse events in advanced breast cancer **(A)** summary AE、skin AEs and systemic AEs **(B)** hematological AEs **(C)** gastrointestinal AEs **(D)** Liver AEs and pain-related AEs **(E)** Circulatory AEs and other types AEs (n = 2).

Twenty-two MAs reported on the AEs of CDK4/6 inhibitor combined with ET in patients with ABC, pooling the most comprehensive data and high-quality evidence for each AE (Figure 6, Supplementary Data S5). Nine studies were examined for summary AEs (Deng et al., 2018; Messina et al., 2018; Ramos-Esquivel et al., 2018; Shimoi et al., 2020; Zheng et al., 2020; Li et al., 2021; Tian et al., 2021). The results showed that the combination of CDK4/6 inhibitor and ET significantly increased the incidence of all-grade AEs and grade 3 or higher AEs in postmenopausal, bone metastasis-only, or ABC patients. Despite the overall benefit of CDK4/6 inhibitors combined with ET for AEs, Ramos-Esquivel A et al. reported more favorable outcomes for CDK4/6 inhibitors combined with fulvestrant, indicating that while the number of serious AEs was higher in the combination group, the advantage ratio for any serious AE was not statistically significant (OR = 1.51.95% CI: 0.74–3.08, *p* = 0.26), and the evidence quality was low (Ramos-Esquivel et al., 2020).

##### 3.4.2.2 AEs of any grade

In the category of any-grade AEs, the incidence of hematologic and gastrointestinal AEs significantly increased when CDK4/6 inhibitors were combined with ET. Notably, significant increases were observed in Neutropenia (n = 9), Leukopenia (n = 8), Anemia (n = 8), Thrombocytopenia (n = 3), Febrile Neutropenia (n = 1), Diarrhea (n = 6), Fatigue (n = 6), Alopecia (n = 3), and VTE (n = 2). However, the association between nausea, vomiting, and decreased appetite in the gastrointestinal tract with combination therapy showed mixed results. Li JM(Li et al., 2020), Xu ZH (Xu et al., 2020), and Yang L (Yang et al., 2021) found no significant difference in nausea and vomiting between the two treatment groups. In contrast, six and three additional studies, respectively, concluded that CDK4/6 inhibitors significantly increased the incidence of nausea and vomiting. Furthermore, Shohdy KS (Shohdy et al., 2017) analyzed 1,350 patients from three clinical trials and observed a significant increase in decreased appetite with combination therapy, while Yang L analyzed 3,685 breast cancer patients from six clinical trials and reported no significant difference between the groups (Yang et al., 2021).

##### 3.4.2.3 Grade 3/4 AEs

In the category of grade 3/4 AEs, hematologic, gastrointestinal, and systemic AEs were prevalent in the CDK4/6 inhibitor group combined with ET. Among hematologic AEs, grade 3/4 neutropenia (n = 8), leukopenia (n = 7), anemia (n = 6), and thrombocytopenia (n = 3) were significantly more common in the combination treatment than in the ET group alone. Notably, GUO LH (Guo et al., 2019) was more controversial regarding the grade 3/4 Fatigue outcome in systemic AEs. Guo LH (Guo et al., 2019) reported no significant risk of grade 3/4 fatigue in the combination therapy group, while six other studies indicated a higher risk (Lasheen et al., 2017; Martel et al., 2018; Toss et al., 2019; Li et al., 2020; Lin et al., 2020; Tian et al., 2021). Interestingly, diarrhea, which showed a significant increase in any-grade AEs, did not significantly differ in grade 3/4 AEs (Shohdy et al., 2017; Toss et al., 2019; Li et al., 2020; Lin et al., 2020; Tian et al., 2021).

##### 3.4.2.4 Subgroup analysis

Seven studies conducted a subgroup analysis of AEs by CDK4/6 inhibitor type (Lasheen et al., 2017; Kassem et al., 2018; Ramos-Esquivel et al., 2018; Li et al., 2020; Lin et al., 2020; Ramos-Esquivel et al., 2020; Tian et al., 2021) Palbociclib exhibited increased hematologic toxicity, particularly in grade 3/4 neutropenia (n = 2), anemia (n = 1), and thrombocytopenia, while ribociclib was more associated with grade 3/4 neutropenia (n = 2), prolonged QT interval (n = 2), and alopecia (n = 1). Abemaciclib was closely linked to diarrhea (n = 4) and elevated blood creatinine (n = 1).

##### 3.4.2.5 Dose reduction and drug withdrawal due to AEs

The likelihood of drug toxicity necessitating therapeutic dose reduction and cessation is a crucial aspect of drug safety assessment. In a meta-analysis by Kassem L, the rate of dose reduction in the CDK4/6 inhibitor group varied from 31.6% to 53.9%, and discontinuation rates due to toxicity ranged from 2.6% to 19.6% (Kassem et al., 2018). Most dose-limiting toxicities were hematologic AEs, although clarity is lacking on whether AEs like fatigue, stomatitis (Lasheen et al., 2017), and gastrointestinal toxicity (Shohdy et al., 2017) can be managed through dose adjustment or discontinuation.

### 3.5 Evaluation of the quality of evidence for AE outcomes

GRADE provides a framework for authors of systematic reviews and health technology assessments to rate the certainty of their evidence (Zeng et al., 2021). This study summarized 13 AE outcomes for two EBCs and 158 AE outcomes for 22 ABC studies. The evidence quality for EBC was predominantly low (4 items, about 30.77%) and very low (9 items, about 69.23%), as assessed using GRADE. The highest level of evidence in ABC was for the venous thromboembolism (VTE) outcome (Thein et al., 2020), Moderate quality evidence was found in five studies (Deng et al., 2018), Tian Q (Tian et al., 2021), Xu ZH (Xu et al., 2020), Li J (Li et al., 2020), Toss A (Toss et al., 2019) addressing various AEs including all-grade AEs, grade 3/4 AEs, leukopenia, grade 3/4 leukopenia, neutropenia, grade 3/4 neutropenia, anemia, grade 3/4 anemia, grade 3/4 diarrhea, and nausea, among others (20 items, about 12.66%). The remaining evidence was categorized as low quality (70 items, about 44.30%) and very low quality (67 items, about 42.40%). Overall, the reduced quality of evidence was largely attributed to inconsistency, risk of bias, and publication bias (Supplementary Data S5).

## 4 Discussion

MA and SR have emerged as crucial supports for clinical decision-making, representing the highest level of evidence in the hierarchy of evidence-based medicine. Conducting effective quality evaluations is essential for the efficient utilization of MA and SR (Gelardi et al., 2021). The meticulous summarization and quality assessment of the clinical AEs of CDK4/6 inhibitors in combination with ET in this study aimed to enhance clinical decision-making, monitoring, and management, thereby improving patient compliance and clinical outcomes.

### 4.1 Main findings

Our review identified 24 MAs and SRs evaluating AEs in 13 EBC cases and 158 ABC cases. Our findings indicated that AEs associated with CDK4/6 inhibitors spanned nine disease areas, including summary AEs, hematologic AEs, gastrointestinal AEs, systemic AEs, liver AEs, circulatory AEs, skin AEs, pain-related AEs, and other AEs. In HR+/HER2- EBC patients, CDK4/6 inhibitor addition significantly correlated with all-grade AEs and grade 3/4 AEs, and notably increased early treatment discontinuation risk. Subgroup analysis showed that EBC AEs were independent of whether palbociclib or abemaciclib was used. For HR+/HER2- ABC patients, combined therapy significantly elevated AEs across all grades, including grade 3/4 leukopenia, neutropenia, anemia, diarrhea, nausea, constipation, fatigue, pyrexia, VTE, abdominal pain, and cough. However, their safety profile was considered acceptable, with evidence quality mostly high to medium. Subgroup analysis of CDK4/6 inhibitor types in ABC revealed that palbociclib was associated with greater hematologic toxicity, especially grade 3/4 neutropenia, anemia, and thrombocytopenia, while ribociclib was linked mainly to grade 3/4 neutropenia, QT interval prolongation, and alopecia. Abemaciclib is closely associated with diarrhea and elevated blood creatinine.

With the increasing use of CDK4/6 inhibitors combined with ET in first and second-line clinical settings, anticipating AEs' risk, timely diagnosis, and management are pivotal in enhancing patients' quality of life (Martel et al., 2018). Despite a high incidence of hematologic, gastrointestinal, and systemic AEs in both EBC and ABC, most were reversible and manageable. Neutropenia was the most common hematologic toxicity, particularly with palbociclib and ribociclib, but it led to febrile neutropenia or infection at much lower rates than chemotherapy. The mechanism of CDK4/6 inhibitors involves reversible blocking of neutrophil precursor cycles, inhibiting proliferation, which is reversible upon discontinuation and tends to lessen over time (Kassem et al., 2018). Thus, clinical applications require monitoring of complete blood counts and timely intervention through discontinuation, dose adjustment, or symptomatic treatment based on individual safety and tolerability (Thill and Schmidt, 2018). Gastrointestinal AEs, such as diarrhea and nausea, especially with abemaciclib, did not significantly increase serious gastrointestinal risk. This may be attributed to abemaciclib’s CDK9 inhibitory effect (Marra and Curigliano, 2019). As diarrhea is typically short-lived and of low severity, it can be managed through dose adjustment or antidiarrheal medications like loperamide. Fatigue, significantly increased in any grade and grade 3/4 AEs, poses a challenge in systemic AEs due to its vague and multidimensional nature, making identification of contributing factors and mitigation measures difficult (Lasheen et al., 2017).

Significantly, the findings related to nausea and vomiting associated with CDK4/6 inhibitors were inconsistent across different AE categories. This inconsistency may stem from variations in the types of CDK4/6 inhibitors used in clinical trials, their modes of administration, differences in study subjects, and the range of clinical trials included in the SRs. Consequently, further research is necessary to reach definitive conclusions. Additionally, we noted substantial heterogeneity in some outcomes. Fundamental aspects of the included studies, such as study population, design, intervention/control measures (dose), follow-up activities, analysis procedures, and outcome indicators, were not adequately detailed, and limited raw data might have contributed to this heterogeneity.

The clinical selection of the three CDK4/6 inhibitors presents a challenging issue, primarily due to the absence of distinct clinical case characteristics, predictive biomarkers, and comparable clinical benefits in PFS and OS(Agostinetto et al., 2021; Gao et al., 2021; Munzone et al., 2021; Falato et al., 2023). A subgroup analysis focusing on CDK4/6 inhibitor types in ABC aimed to address this issue. Palbociclib was associated with increased neutropenia, anemia, and thrombocytopenia, while ribociclib was linked mainly to neutropenia, prolonged QT interval, and alopecia. Abemaciclib was strongly correlated with diarrhea and elevated blood creatinine. This suggests that patients with ABC who have hematological disorders or tendencies may be better suited for abemaciclib, while those with gastrointestinal disorders and renal dysfunction or tendencies might be more appropriate for ribociclib or palbociclib. Furthermore, patients with cardiac disorders or tendencies might be more suitable for ribociclib or abemaciclib. This could be a significant opportunity to improve patient quality of life, advance clinical decision-making accuracy, and achieve personalized precision medicine.

### 4.2 Strengths and limitations

To our knowledge, this is the first to use an umbrella review of studies to summarize CDK4/6 inhibitors combined with ET for HR+/HER2-breast cancer AEs. This approach mitigates the bias inherent in individual MAs/SRs and enhances the accuracy of study outcomes. Second, we included MAs/SRs that used only RCT study types and excluded literature that used single-arm studies, and non-randomized prospective, retrospective, and observational studies as study types, which further reduced the interference of subjective factors and improved the scientific validity of the findings. In Additionally, this review employed the recently published PRISMA 2020 guidelines for reporting quality assessment (Sohrabi et al., 2021), a significant update over the PRISMA 2009 guidelines typically referenced in most MAs/SRs (Shea et al., 2009). The PRISMA 2020 guidelines offer improvements in areas like data items, data synthesis methods, study outcome selection, and results synthesis. These updated guidelines facilitate the generation of high-quality evidence that can support clinical decision-making and practice (Page et al., 2021).

This review, however, is not without limitations. Firstly, despite employing the latest versions of AMSTAR-2, PRISMA 2020, and GRADE for quality assessment of the selected literature, it is important to note that all three scales are inherently subjective, which could introduce bias to our findings. To mitigate this, two researchers independently conducted the assessments, with any differences resolved through consensus by a third-party reviewer (CHH), thereby aiming to maximize the accuracy of our evaluation. Moreover, the MAs/SRs included in this study were based on global multicenter RCTs, potentially leading to publication bias and a diminished quality of evidence due to the limited number of RCTs.

## 5 Conclusion

In patients with HR+/HER2- EBC, the addition of CDK4/6 inhibitors significantly increased the incidence of all-grade AEs and grade 3/4 AEs compared to ET alone, along with a notable rise in the risk of early treatment discontinuation. In patients with HR+/HER2- ABC, palbociclib was associated with increased hematologic toxicity, primarily grade 3/4 neutropenia, anemia, and thrombocytopenia, while ribociclib was linked mainly to grade 3/4 neutropenia, QT interval prolongation, and alopecia. Abemaciclib was closely related to diarrhea and elevated blood creatinine. Despite these findings, the safety of these inhibitors was deemed acceptable, and the overall quality of evidence was mostly moderate. The comprehensive summary and evaluation of these AEs will aid in the selection of tailored and precise treatments based on the history of breast cancer patients. Furthermore, it will assist clinicians in effectively anticipating, diagnosing, and managing AEs associated with CDK4/6 inhibitors, ultimately enhancing patients' quality of life.

## Data Availability

The original contributions presented in the study are included in the article/Supplementary Material, further inquiries can be directed to the corresponding author.
