# Supplementary material for: The adverse events of CDK4/6 inhibitors for HR+/ HER2- breast cancer: an umbrella review of meta-analyses of randomized controlled trials
Source: Front Pharmacol. 2024 Jan 15;15:1269922. doi: 10.3389/fphar.2024.1269922 (PMC10823006; doi:10.3389/fphar.2024.1269922)
Supplement: Supplementary file 1 [file DataSheet1.docx]

Supplementary Material

The Adverse Events of CDK4/6 Inhibitors: An Umbrella Re-view of Meta-Analyses of Randomized Controlled Trials

**Dongqing Pu^1^, Yue Wu^2^, Debo Xu^2^, Guangxi Shi^3^, Hanhan Chen^3^, Dandan Feng^1^, Mengdi Zhang^1^, Jingwei Li^3*^**

^1^Department of First Clinical Medical College, Shandong University of Traditional Chinese Medicine, Shandong, China. ^2^Department of College of Traditional Chinese Medicine, Shandong University of Traditional Chinese Medicine, Shandong, China. ^3^Department of Breast Thyroid Surgery, Affiliated Hospital of Shandong University of Traditional Chinese Medicine, Shandong, China

*** Correspondence:**Corresponding Author: Jingwei Li
71000395@sdutcm.edu.cn

Contents

[1 Supplementary table S1. Electronic Database Search Strategies. 3](#_Toc141693436)

[1.1 For COCHRANE 3](#_Toc141693437)

[1.2 For PUBMED 3](#_Toc141693438)

[1.3 For EMBASE 4](#_Toc141693439)

[1.4 For Web of science 5](#_Toc141693440)

[2 Supplementary table S2 The AMSTAR-2、Prisma 2020 and GRADE scales 6](#_Toc141693441)

[2.1 The AMSTAR-2 scale 6](#_Toc141693442)

[2.2 The PRISMA 2020 Checklist 13](#_Toc141693443)

[2.3 The GRADEpro GDT scale 16](#_Toc141693444)

[3 Supplementary table S3 Quality appraisal results of included systematic reviews using the AMSTAR-2 Tool. 17](#_Toc141693445)

[4 Supplementary table S4. Quality appraisal results of included systematic reviews using the PRISMA 2020. 19](#_Toc141693446)

[5 Supplementary table S5 Clinical Outcomes in Patients with Breast Cancer and GRADE Scale 23](#_Toc141693447)

[5.1 Early breast cancer 23](#_Toc141693448)

[5.2 Advance breast cancer 26](#_Toc141693449)

# Supplementary table S1. Electronic Database Search Strategies.

## For COCHRANE

| #1 | MeSH descriptor: [Breast Neoplasms] explode all trees |
| --- | --- |
| #2 | (breast cancer*):ti,ab,kw |
| #3 | (breast neoplasm*):ti,ab,kw |
| #4 | (breast carcinoma*):ti,ab,kw |
| #5 | (breast tumour*):ti,ab,kw |
| #6 | (breast tumor*):ti,ab,kw |
| #7 | #1 OR #2 OR #3 OR #4 OR #5 OR #6 |
| #8 | (Cyclin-Dependent Kinases 4 and 6 Inhibitor*):ti,ab,kw |
| #9 | (CDK4/6 inhibit*):ti,ab,kw |
| #10 | (CDK4/6i*):ti,ab,kw |
| #11 | (Palbociclib):ti,ab,kw |
| #12 | (Ribociclib):ti,ab,kw |
| #13 | (Abemaciclib):ti,ab,kw |
| #14 | (Dalpiciclib):ti,ab,kw |
| #15 | #8 OR #9 OR #10 OR #11 OR #12 OR #13 OR #14 |
| #16 | MeSH descriptor: [Meta-Analysis as Topic] explode all trees |
| #17 | MeSH descriptor: [Systematic Review as Topic] explode all trees |
| #18 | (Meta-Analysis):ti,ab,kw |
| #19 | (Meta Analysis):ti,ab,kw |
| #20 | (Meta-Analyses):ti,ab,kw |
| #21 | (Metaanalysis):ti,ab,kw |
| #22 | (systematic review):ti,ab,kw |
| #23 | #16 OR #17 OR #18 OR #19 OR #20 OR #21 OR #22 |
| #24 | #7 AND #15 AND #23 |

## For PUBMED

| #1 | “Breast Neoplasms” [MeSH] |
| --- | --- |
| #2 | breast [Title/Abstract] |
| #3 | cancer*[Title/Abstract] OR tumour*[Title/Abstract] OR tumor*[Title/Abstract] OR neoplas*[Title/Abstract] OR carcinoma*[Title/Abstract] |
| #4 | #2 AND #3 |
| #5 | #1 OR #4 |
| #6 | “Cyclin-Dependent Kinases 4 and 6 Inhibitor*” [Title/Abstract] |
| #7 | CDK4/6 inhibit* [Title/Abstract] |
| #8 | CDK4/6i* [Title/Abstract] |
| #9 | Palbociclib [Title/Abstract] |
| #10 | Ribociclib [Title/Abstract] |
| #11 | Abemaciclib [Title/Abstract] |
| #12 | Dalpiciclib [Title/Abstract] |
| #13 | #6 OR #7 OR #8 OR #9 OR #10 OR #11 OR #12 |
| #14 | “Meta-Analysis as Topic” [MeSH] |
| #15 | “Systematic Review as Topic” [MeSH] |
| #16 | meta-analysis [Title/Abstract] |
| #17 | meta analysis [Title/Abstract] |
| #18 | meta-analyses [Title/Abstract] |
| #19 | Metaanalysis [Title/Abstract] |
| #20 | systematic review [Title/Abstract] |
| #21 | #14 OR #15 OR #16 OR #17 OR #18 OR #19 OR #20 |
| #21 | #5 AND #13 AND #21 |

## For EMBASE

| #1 | ‘breast tumor’/exp OR ‘breast cancer*’:ab,ti OR ‘breast neoplasm*’:ab,ti OR ‘breast carcinoma*’:ab,ti OR ‘breast tumour*’:ab,ti OR ‘breast tumor*’:ab,ti |
| --- | --- |
| #2 | ‘Cyclin-Dependent Kinases 4 and 6 Inhibitor*’:ab,ti OR ‘CDK4/6 inhibit*’:ab,ti OR ‘CDK4/6i*’:ab,ti OR ‘Palbociclib’:ab,ti OR ‘Ribociclib’:ab,ti OR ‘Abemaciclib’:ab,ti OR ‘Dalpiciclib’:ab,ti |
| #3 | ‘meta analysis’/exp OR ‘systematic reviews’/exp OR ‘Meta-Analysis as Topic’:ab,ti OR ‘Systematic Review as Topic’:ab,ti OR ‘meta analysis’:ab,ti OR ‘meta analyses’:ab,ti OR ‘metaanalysis’:ab,ti OR ‘systematic review’:ab,ti |
| #4 | #1 AND #2 AND #3 |

## For Web of science

| #1 | TS=(breast cancer*) OR TS=(breast neoplasm*) OR TS=(breast carcinoma*) OR TS=(breast tumour*) OR TS=(breast tumor*) |
| --- | --- |
| #2 | TS=(“Cyclin-Dependent Kinases 4 and 6 Inhibitor*”) OR TS=(“CDK4/6 inhibit*”) OR TS=(Palbociclib) OR TS=(Ribociclib) OR TS=(Abemaciclib) OR TS=(Dalpiciclib) |
| #3 | TS=(Meta-Analysis as Topic) OR TS=(Systematic Review as Topic) OR TS=(meta-analysis) OR TS=(meta analysis) OR TS=(meta analyses) OR TS-(metaanalysis) OR TS=(systematic review) |
| #4 | #1 AND #2 AND #3 |

# Supplementary table S2 The AMSTAR-2、Prisma 2020 and GRADE scales

## The AMSTAR-2 scale

| **1. Did the research questions and inclusion criteria for the review include the components of PICO?** | | | | | | | | |
| --- | --- | --- | --- | --- | --- | --- | --- | --- |
|  | For Yes:   - Population - Intervention - Comparator group - Outcome | | Optional (recommended)   - Timeframe for follow-up | | | - Yes - No | |  |
| **2. Did the report of the review contain an explicit statement that the review methods were established prior to the conduct of the review and did the report justify any significant deviations from the protocol?** | | | | | | | | |
|  | For Partial Yes:  The authors state that they had a written protocol or guide that included ALL the following:   - review question(s) - a search strategy - inclusion/exclusion criteria - a risk of bias assessment | | For Yes:  As for partial yes, plus the protocol should be registered and should also have specified:   - a meta-analysis/synthesis plan, if appropriate, *and* - a plan for investigating causes of heterogeneity - justification for any deviations from the protocol | | | - Yes - Partial Yes - No | |  |
| **3. Did the review authors explain their selection of the study designs for inclusion in the review?** | | | | | | | | |
|  | For Yes, the review should satisfy ONE of the following:   - *Explanation for* including only RCTs - OR *Explanation for* including only NRSI - OR *Explanation for* including both RCTs and NRSI | | | | | - Yes - No | |  |
| **4. Did the review authors use a comprehensive literature search strategy?** | | | | | | | | |
|  | For Partial Yes (all the following):   - searched at least 2 databases (relevant to research question) - provided key word and/or search strategy - justified publication restrictions (e.g. language) | | For Yes, should also have (all the following):   - searched the reference lists / bibliographies of included studies - searched trial/study registries - included/consulted content experts in the field - where relevant, searched for grey literature - conducted search within 24 months of completion of the review | | | - Yes - Partial Yes - No | |  |
| **5. Did the review authors perform study selection in duplicate?** | | | | | | | | |
|  | For Yes, either ONE of the following:   - at least two reviewers independently agreed on selection of eligible studies and achieved consensus on which studies to include - OR two reviewers selected a sample of eligible studies and achieved good agreement (at least 80 percent), with the remainder selected by one reviewer. | | | | | - Yes - No | |  |
| **6. Did the review authors perform data extraction in duplicate?** | | | | | | | | |
|  | | For Yes, either ONE of the following:   - at least two reviewers achieved consensus on which data to extract from included studies - OR two reviewers extracted data from a sample of eligible studies and achieved good agreement (at least 80 percent), with the remainder extracted by one reviewer. | | | | | - Yes - No | |
| **7. Did the review authors provide a list of excluded studies and justify the exclusions?** | | | | | | | | |
|  | | For Partial Yes:   - provided a list of all potentially relevant studies that were read in full-text form but excluded from the review | For Yes, must also have:   - Justified the exclusion from the review of each potentially relevant study | | | | - Yes - Partial Yes - No | |
| **8. Did the review authors describe the included studies in adequate detail?** | | | | | | | | |
|  | | For Partial Yes (ALL the following):   - described populations - described interventions - described comparators - described outcomes - described research designs | For Yes, should also have ALL the following:   - described population in detail - described intervention in detail (including doses where relevant) - described comparator in detail (including doses where relevant) - described study’s setting - timeframe for follow-up | | | | - Yes - Partial Yes - No | |
| **9. Did the review authors use a satisfactory technique for assessing the risk of bias (RoB) in individual studies that were included in the review?** | | | | | | | | |
|  | | **RCTs**  For Partial Yes, must have assessed RoB from   - unconcealed allocation, *and* - lack of blinding of patients and assessors when assessing outcomes (unnecessary for objective outcomes such as all- cause mortality) | For Yes, must also have assessed RoB from:   - allocation sequence that was not truly random, *and* - selection of the reported result from among multiple measurements or analyses of a specified outcome | | | | - Yes - Partial Yes - No - Includes only NRSI | |
|  | | **NRSI**  For Partial Yes, must have assessed RoB:   - from confounding, *and* - from selection bias | For Yes, must also have assessed RoB:   - methods used to ascertain exposures and outcomes, *and* - selection of the reported result from among multiple measurements or analyses of a specified outcome | | | | - Yes - Partial Yes - No - Includes only RCTs | |
| **10. Did the review authors report on the sources of funding for the studies included in the review?** | | | | | | | | |
|  | | For Yes   - Must have reported on the sources of funding for individual studies included  Yes in the review. Note: Reporting that the reviewers looked for this information  No but it was not reported by study authors also qualifies | | | | | | |
| **11. If meta-analysis was performed did the review authors use appropriate methods for statistical combination of results?** | | | | | | | | |
|  | **RCTs**  For Yes:   - The authors justified combining the data in a meta-analysis   - AND they used an appropriate weighted technique to combine study results and adjusted for heterogeneity if present.   - AND investigated the causes of any heterogeneity | | | | - Yes - No - No meta-analysis conducted | | |  |
|  | **For NRSI**  For Yes:   - The authors justified combining the data in a meta-analysis   - AND they used an appropriate weighted technique to combine study results, adjusting for heterogeneity if present   - AND they statistically combined effect estimates from NRSI that were adjusted for confounding, rather than combining raw data, or justified combining raw data when adjusted effect estimates were not available   - AND they reported separate summary estimates for RCTs and NRSI separately when both were included in the review | | | | - Yes - No - No meta-analysis conducted | | |  |
| **12. If meta-analysis was performed, did the review authors assess the potential impact of RoB in individual studies on the results of the meta-analysis or other evidence synthesis?** | | | | | | | | |
|  | For Yes:   - included only low risk of bias RCTs - OR, if the pooled estimate was based on RCTs and/or NRSI at variable RoB, the authors performed analyses to investigate possible impact of RoB on summary estimates of effect. | | | | - Yes - No - No meta-analysis conducted | | |  |
| **13. Did the review authors account for RoB in individual studies when interpreting/ discussing the results of the review?** | | | | | | | | |
|  | For Yes:   - included only low risk of bias RCTs - OR, if RCTs with moderate or high RoB, or NRSI were included the review provided a discussion of the likely impact of RoB on the results | | | | - Yes - No | | |  |
| **14. Did the review authors provide a satisfactory explanation for, and discussion of, any heterogeneity observed in the results of the review?** | | | | | | | | |
|  | For Yes:   - There was no significant heterogeneity in the results - OR if heterogeneity was present the authors performed an investigation of sources of any heterogeneity in the results and discussed the impact of this on the results of the review | | | | - Yes - No | | |  |
| **15. If they performed quantitative synthesis did the review authors carry out an adequate investigation of publication bias (small study bias) and discuss its likely impact on the results of the review?** | | | | | | | | |
|  | For Yes:   - performed graphical or statistical tests for publication bias and discussed the likelihood and magnitude of impact of publication bias | | | | - Yes - No - No meta-analysis conducted | | |  |
| **16. Did the review authors report any potential sources of conflict of interest, including any funding they received for conducting the review?** | | | | | | | | |
|  | For Yes:   - The authors reported no competing interests OR - The authors described their funding sources and how they managed potential conflicts of interest | | | - Yes - No | | | |  |

## The PRISMA 2020 Checklist

| **Section and Topic** | **Item #** | **Checklist item** | **Location where item is reported** |
| --- | --- | --- | --- |
| **TITLE** | | |  |
| Title | 1 | Identify the report as a systematic review. |  |
| **ABSTRACT** | | |  |
| Abstract | 2 | See the PRISMA 2020 for Abstracts checklist. |  |
| **INTRODUCTION** | | |  |
| Rationale | 3 | Describe the rationale for the review in the context of existing knowledge. |  |
| Objectives | 4 | Provide an explicit statement of the objective(s) or question(s) the review addresses. |  |
| **METHODS** | | |  |
| Eligibility criteria | 5 | Specify the inclusion and exclusion criteria for the review and how studies were grouped for the syntheses. |  |
| Information sources | 6 | Specify all databases, registers, websites, organisations, reference lists and other sources searched or consulted to identify studies. Specify the date when each source was last searched or consulted. |  |
| Search strategy | 7 | Present the full search strategies for all databases, registers and websites, including any filters and limits used. |  |
| Selection process | 8 | Specify the methods used to decide whether a study met the inclusion criteria of the review, including how many reviewers screened each record and each report retrieved, whether they worked independently, and if applicable, details of automation tools used in the process. |  |
| Data collection process | 9 | Specify the methods used to collect data from reports, including how many reviewers collected data from each report, whether they worked independently, any processes for obtaining or confirming data from study investigators, and if applicable, details of automation tools used in the process. |  |
| Data items | 10a | List and define all outcomes for which data were sought. Specify whether all results that were compatible with each outcome domain in each study were sought (e.g. for all measures, time points, analyses), and if not, the methods used to decide which results to collect. |  |
|  | 10b | List and define all other variables for which data were sought (e.g. participant and intervention characteristics, funding sources). Describe any assumptions made about any missing or unclear information. |  |
| Study risk of bias assessment | 11 | Specify the methods used to assess risk of bias in the included studies, including details of the tool(s) used, how many reviewers assessed each study and whether they worked independently, and if applicable, details of automation tools used in the process. |  |
| Effect measures | 12 | Specify for each outcome the effect measure(s) (e.g. risk ratio, mean difference) used in the synthesis or presentation of results. |  |
| Synthesis methods | 13a | Describe the processes used to decide which studies were eligible for each synthesis (e.g. tabulating the study intervention characteristics and comparing against the planned groups for each synthesis (item #5)). |  |
|  | 13b | Describe any methods required to prepare the data for presentation or synthesis, such as handling of missing summary statistics, or data conversions. |  |
|  | 13c | Describe any methods used to tabulate or visually display results of individual studies and syntheses. |  |
|  | 13d | Describe any methods used to synthesize results and provide a rationale for the choice(s). If meta-analysis was performed, describe the model(s), method(s) to identify the presence and extent of statistical heterogeneity, and software package(s) used. |  |
|  | 13e | Describe any methods used to explore possible causes of heterogeneity among study results (e.g. subgroup analysis, meta-regression). |  |
|  | 13f | Describe any sensitivity analyses conducted to assess robustness of the synthesized results. |  |
| Reporting bias assessment | 14 | Describe any methods used to assess risk of bias due to missing results in a synthesis (arising from reporting biases). |  |
| Certainty assessment | 15 | Describe any methods used to assess certainty (or confidence) in the body of evidence for an outcome. |  |
| **RESULTS** | | |  |
| Study selection | 16a | Describe the results of the search and selection process, from the number of records identified in the search to the number of studies included in the review, ideally using a flow diagram. |  |
|  | 16b | Cite studies that might appear to meet the inclusion criteria, but which were excluded, and explain why they were excluded. |  |
| Study characteristics | 17 | Cite each included study and present its characteristics. |  |
| Risk of bias in studies | 18 | Present assessments of risk of bias for each included study. |  |
| Results of individual studies | 19 | For all outcomes, present, for each study: (a) summary statistics for each group (where appropriate) and (b) an effect estimate and its precision (e.g. confidence/credible interval), ideally using structured tables or plots. |  |
| Results of syntheses | 20a | For each synthesis, briefly summarise the characteristics and risk of bias among contributing studies. |  |
|  | 20b | Present results of all statistical syntheses conducted. If meta-analysis was done, present for each the summary estimate and its precision (e.g. confidence/credible interval) and measures of statistical heterogeneity. If comparing groups, describe the direction of the effect. |  |
|  | 20c | Present results of all investigations of possible causes of heterogeneity among study results. |  |
|  | 20d | Present results of all sensitivity analyses conducted to assess the robustness of the synthesized results. |  |
| Reporting biases | 21 | Present assessments of risk of bias due to missing results (arising from reporting biases) for each synthesis assessed. |  |
| Certainty of evidence | 22 | Present assessments of certainty (or confidence) in the body of evidence for each outcome assessed. |  |
| **DISCUSSION** | | |  |
| Discussion | 23a | Provide a general interpretation of the results in the context of other evidence. |  |
|  | 23b | Discuss any limitations of the evidence included in the review. |  |
|  | 23c | Discuss any limitations of the review processes used. |  |
|  | 23d | Discuss implications of the results for practice, policy, and future research. |  |
| **OTHER INFORMATION** | | |  |
| Registration and protocol | 24a | Provide registration information for the review, including register name and registration number, or state that the review was not registered. |  |
|  | 24b | Indicate where the review protocol can be accessed, or state that a protocol was not prepared. |  |
|  | 24c | Describe and explain any amendments to information provided at registration or in the protocol. |  |
| Support | 25 | Describe sources of financial or non-financial support for the review, and the role of the funders or sponsors in the review. |  |
| Competing interests | 26 | Declare any competing interests of review authors. |  |
| Availability of data, code and other materials | 27 | Report which of the following are publicly available and where they can be found: template data collection forms; data extracted from included studies; data used for all analyses; analytic code; any other materials used in the review. |  |

## The GRADEpro GDT scale

| Certainty assessment | | | | | | |
| --- | --- | --- | --- | --- | --- | --- |
| Participant(studies) | Risk of bias | inconsistency | indirectness | accuracy | publication bias | Overall certainty of evidence |

# Supplementary table S3 Quality appraisal results of included systematic reviews using the AMSTAR-2 Tool.

|  | Agostinetto, et al.2021 | Gao,et al.2021 | GUO,et al.2019 | Lee,et al.2019 | Li,et al.2020 | Li,et al.2020 | Li,et al.2021 | Lin,et al.2020 | Messina,et al.2018 | Ramos-Esquivel,et al.2020 | Ramos-Esquivel,et al.2018 | Shimoi,et al.2020 | Tian,et al.2021 | Xu,et al.2020 | Yang,et al.2021 | Zheng,et al.2020 | Kassem,et al.2018 | Lasheen,et al.2017 | Shohdy,et al.2017 | Thein,et al.2020 | Martel,et al.2017 | Toss,et al.2019 | Deng,et al.2018 | Bolzacchini,et al.2021 | Summary Y | Summary N | Summary PY |
| --- | --- | --- | --- | --- | --- | --- | --- | --- | --- | --- | --- | --- | --- | --- | --- | --- | --- | --- | --- | --- | --- | --- | --- | --- | --- | --- | --- |
| Q1 | Y | Y | Y | Y | Y | Y | Y | Y | Y | Y | Y | Y | Y | Y | Y | Y | Y | Y | Y | Y | Y | Y | Y | Y | 24 | 0 | 0 |
| Q2 | Y | Y | N | N | N | N | N | N | N | N | N | N | N | N | Y | N | N | N | N | N | Y | Y | N | Y | 6 | 18 | 0 |
| Q3 | Y | Y | Y | Y | Y | Y | Y | Y | Y | Y | Y | Y | Y | Y | Y | Y | Y | Y | Y | Y | Y | Y | Y | Y | 24 | 0 | 0 |
| Q4 | Y | Y | Y | Y | Y | Y | Y | Y | Y | Y | Y | Y | Y | PY | Y | Y | PY | PY | Y | Y | Y | PY | Y | Y | 20 | 0 | 4 |
| Q5 | Y | Y | Y | N | Y | Y | Y | Y | Y | N | Y | N | Y | N | Y | Y | Y | Y | N | N | Y | Y | N | Y | 17 | 7 | 0 |
| Q6 | Y | Y | Y | Y | Y | Y | Y | Y | N | N | Y | Y | Y | Y | Y | Y | Y | Y | Y | Y | Y | Y | Y | Y | 22 | 2 | 0 |
| Q7 | N | N | N | N | N | N | N | N | N | N | N | N | N | N | N | N | N | N | N | N | N | N | N | N | 0 | 24 | 0 |
| Q8 | PY | PY | PY | PY | PY | PY | PY | Y | Y | PY | PY | PY | PY | PY | PY | Y | PY | PY | PY | PY | PY | PY | PY | PY | 3 | 0 | 21 |
| Q9 | Y | Y | Y | N | Y | N | Y | Y | Y | Y | Y | Y | Y | Y | Y | Y | N | N | Y | Y | N | N | Y | Y | 18 | 6 | 0 |
| Q10 | N | N | N | N | N | N | N | N | N | Y | Y | N | N | N | N | N | N | N | N | N | N | N | N | N | 2 | 22 | 0 |
| Q11 | Y | Y | Y | Y | Y | Y | Y | Y | Y | Y | Y | Y | Y | Y | Y | Y | Y | Y | Y | Y | Y | Y | Y | Y | 24 | 0 | 0 |
| Q12 | Y | Y | Y | N | Y | Y | Y | Y | Y | Y | Y | N | Y | Y | Y | Y | N | N | N | Y | N | N | Y | N | 16 | 8 | 0 |
| Q13 | Y | N | N | N | Y | N | Y | N | Y | Y | Y | Y | N | Y | Y | Y | N | N | N | Y | N | N | Y | Y | 13 | 11 | 0 |
| Q14 | Y | Y | N | N | N | N | N | N | N | Y | N | N | N | N | N | N | N | N | N | N | Y | N | N | N | 4 | 20 | 0 |
| Q15 | Y | N | N | N | Y | N | N | Y | N | Y | Y | Y | Y | Y | Y | N | N | N | N | Y | N | Y | Y | N | 12 | 12 | 0 |
| Q16 | Y | Y | N | Y | Y | N | Y | Y | Y | Y | Y | Y | Y | Y | Y | Y | Y | Y | Y | Y | Y | Y | N | Y | 21 | 3 | 0 |
| Quality evaluation | L | VL | VL | VL | VL | VL | VL | VL | VL | VL | VL | VL | VL | VL | L | VL | VL | VL | VL | VL | VL | VL | VL | VL |  |  |  |

Y, Yes; PY, Partial Yes; N, No. L, Low; VL, Very low.

# Supplementary table S4. Quality appraisal results of included systematic reviews using the PRISMA 2020.

|  | Agostinetto et al.2021 | Gao et al.2021 | Guo et al.2019 | Lee et al.2019 | Li et al.2020 | Li et al. 2020 | Li et al.2021 | Lin et al.2020 | Messina et al.2018 | Ramos-Esquivel et al.2020 | Ramos-Esquivel et al.2018 | Shimoi et al.2020 | Tian et al.2021 | Xu et al.2020 | Yang et al.2021 | Zheng et al.2020 | Kassem et al.2018 | Lasheen et al.2017 | Shohdy et al.2017 | Thein et al.2020 | Martel et al.2017 | Toss et al.2019 | Deng et al.2018 | Bolzacchini et al.2021 |
| --- | --- | --- | --- | --- | --- | --- | --- | --- | --- | --- | --- | --- | --- | --- | --- | --- | --- | --- | --- | --- | --- | --- | --- | --- |
| Item-1 | 1 | 1 | 1 | 1 | 1 | 1 | 1 | 0 | 1 | 1 | 1 | 1 | 1 | 1 | 1 | 1 | 1 | 1 | 1 | 1 | 1 | 1 | 1 | 1 |
| Item-2 | 1 | 0.5 | 0.5 | 0.5 | 0.5 | 0.5 | 0.5 | 0.5 | 0.5 | 0.5 | 0.5 | 0.5 | 0.5 | 0.5 | 1 | 0.5 | 0.5 | 0.5 | 0.5 | 0.5 | 0.5 | 0.5 | 0.5 | 0.5 |
| Item-3 | 1 | 1 | 1 | 1 | 1 | 1 | 1 | 1 | 1 | 1 | 1 | 1 | 1 | 1 | 1 | 1 | 1 | 1 | 1 | 1 | 1 | 1 | 1 | 1 |
| Item-4 | 1 | 1 | 1 | 1 | 1 | 1 | 1 | 1 | 1 | 1 | 1 | 1 | 1 | 1 | 1 | 1 | 1 | 1 | 1 | 1 | 1 | 1 | 1 | 1 |
| Item-5 | 1 | 1 | 1 | 0 | 1 | 1 | 0.5 | 1 | 1 | 0.5 | 1 | 1 | 1 | 0.5 | 1 | 1 | 1 | 1 | 1 | 0.5 | 1 | 1 | 1 | 0.5 |
| Item-6 | 1 | 1 | 1 | 1 | 1 | 1 | 1 | 1 | 1 | 1 | 1 | 1 | 1 | 1 | 1 | 1 | 1 | 1 | 1 | 1 | 1 | 1 | 1 | 1 |
| Item-7 | 0 | 0.5 | 0 | 0 | 0.5 | 0 | 0.5 | 1 | 0 | 0 | 0.5 | 0 | 0 | 0 | 0 | 0.5 | 0 | 0 | 0 | 0 | 0 | 0 | 0.5 | 0 |
| Item-8 | 1 | 1 | 1 | 0 | 1 | 1 | 1 | 1 | 1 | 1 | 1 | 1 | 1 | 1 | 1 | 1 | 1 | 1 | 1 | 1 | 1 | 1 | 1 | 1 |
| Item-9 | 1 | 1 | 1 | 1 | 1 | 1 | 1 | 1 | 1 | 0 | 1 | 1 | 1 | 1 | 1 | 1 | 1 | 1 | 1 | 1 | 1 | 1 | 1 | 1 |
| Item-10a | 1 | 1 | 1 | 1 | 1 | 1 | 1 | 1 | 1 | 1 | 1 | 1 | 1 | 1 | 1 | 1 | 1 | 1 | 1 | 1 | 1 | 1 | 1 | 1 |
| Item-10b | 0.5 | 0.5 | 0.5 | 0.5 | 0.5 | 0.5 | 0.5 | 0.5 | 0.5 | 0.5 | 0.5 | 0.5 | 0.5 | 0.5 | 0.5 | 0.5 | 0.5 | 0.5 | 0.5 | 0.5 | 0.5 | 0.5 | 0.5 | 0.5 |
| Item-11 | 1 | 1 | 1 | 0 | 1 | 0 | 1 | 1 | 1 | 1 | 1 | 1 | 1 | 1 | 1 | 1 | 0 | 0 | 1 | 0.5 | 0 | 0 | 1 | 1 |
| Item-12 | 1 | 1 | 1 | 1 | 1 | 1 | 1 | 1 | 1 | 1 | 1 | 1 | 1 | 1 | 1 | 1 | 1 | 1 | 1 | 1 | 1 | 1 | 1 | 1 |
| Item-13a | 1 | 1 | 1 | 1 | 1 | 1 | 1 | 1 | 1 | 1 | 1 | 0 | 1 | 1 | 1 | 1 | 0 | 0 | 0 | 0 | 0 | 0 | 0 | 0 |
| Item-13b | 1 | 1 | 1 | 1 | 1 | 1 | 1 | 1 | 1 | 1 | 1 | 0 | 1 | 1 | 1 | 1 | 1 | 1 | 1 | 1 | 1 | 0 | 0 | 1 |
| Item-13c | 0 | 1 | 1 | 0 | 1 | 1 | 1 | 1 | 1 | 1 | 1 | 1 | 1 | 1 | 1 | 1 | 1 | 1 | 1 | 1 | 1 | 1 | 1 | 1 |
| Item-13d | 1 | 1 | 1 | 1 | 1 | 1 | 1 | 1 | 1 | 1 | 1 | 1 | 1 | 1 | 1 | 1 | 1 | 1 | 1 | 1 | 1 | 1 | 1 | 1 |
| Item-13e | 0 | 1 | 0 | 1 | 0 | 0 | 0 | 1 | 0 | 0 | 0 | 0 | 0 | 1 | 0 | 1 | 1 | 1 | 1 | 1 | 0 | 0 | 0 | 1 |
| Item-13f | 1 | 1 | 0 | 0 | 0 | 0 | 0 | 0 | 0 | 0 | 0 | 0 | 0 | 0 | 0 | 1 | 0 | 0 | 0 | 0 | 0 | 0 | 0 | 0 |
| Item-14 | 1 | 0 | 0 | 0 | 1 | 0 | 0 | 1 | 0 | 1 | 1 | 1 | 1 | 1 | 1 | 0 | 0 | 0 | 0 | 1 | 0 | 1 | 1 | 0 |
| Item-15 | 1 | 1 | 0 | 0.5 | 0.5 | 0 | 0 | 0 | 0 | 0 | 0 | 0 | 0.5 | 0 | 0 | 0.5 | 0 | 0 | 0 | 0.5 | 0 | 0 | 0 | 0 |
| Item-16a | 1 | 1 | 1 | 1 | 1 | 1 | 1 | 1 | 1 | 1 | 1 | 1 | 1 | 1 | 1 | 1 | 1 | 1 | 1 | 1 | 1 | 1 | 1 | 1 |
| Item-16b | 0 | 0 | 0 | 0 | 0 | 0 | 0 | 0 | 0 | 0 | 0 | 0 | 0 | 0 | 0 | 0 | 0 | 0 | 0 | 0 | 0 | 0 | 0 | 0 |
| Item-17 | 1 | 1 | 1 | 1 | 1 | 1 | 1 | 1 | 1 | 1 | 1 | 1 | 1 | 1 | 1 | 1 | 1 | 1 | 1 | 1 | 1 | 1 | 1 | 1 |
| Item-18 | 1 | 1 | 1 | 0 | 1 | 0 | 1 | 1 | 1 | 1 | 1 | 1 | 1 | 1 | 1 | 1 | 0 | 0 | 1 | 0.5 | 0 | 0 | 1 | 1 |
| Item-19 | 1 | 1 | 1 | 1 | 1 | 1 | 1 | 1 | 1 | 1 | 1 | 1 | 1 | 1 | 1 | 1 | 1 | 1 | 1 | 1 | 1 | 1 | 1 | 1 |
| Item-20a | 0.5 | 0.5 | 0.5 | 0.5 | 0.5 | 0.5 | 0.5 | 0.5 | 0.5 | 0.5 | 0.5 | 0.5 | 0.5 | 0.5 | 0.5 | 0.5 | 0.5 | 0.5 | 0.5 | 0.5 | 0.5 | 0.5 | 0.5 | 0.5 |
| Item-20b | 1 | 1 | 1 | 1 | 1 | 1 | 1 | 1 | 1 | 1 | 1 | 1 | 1 | 1 | 1 | 1 | 1 | 1 | 1 | 1 | 1 | 1 | 1 | 1 |
| Item-20c | 1 | 1 | 0 | 1 | 1 | 0 | 0 | 1 | 0 | 1 | 1 | 0 | 1 | 1 | 1 | 1 | 1 | 1 | 1 | 1 | 0 | 1 | 1 | 0 |
| Item-20d | 1 | 1 | 0 | 0 | 0 | 0 | 0 | 0 | 0 | 0 | 0 | 0 | 0 | 0 | 0 | 0 | 0 | 0 | 0 | 0 | 0 | 0 | 0 | 0 |
| Item-21 | 1 | 0 | 0 | 0 | 1 | 0 | 0 | 1 | 0 | 1 | 1 | 1 | 1 | 1 | 1 | 0 | 0 | 0 | 0 | 1 | 0 | 1 | 1 | 0 |
| Item-22 | 1 | 1 | 0 | 0.5 | 0.5 | 0 | 0 | 0 | 0 | 0 | 0 | 0 | 0.5 | 0 | 0 | 0.5 | 0 | 0 | 0 | 0.5 | 0 | 0 | 0 | 0 |
| Item-23a | 1 | 1 | 1 | 1 | 1 | 1 | 1 | 1 | 1 | 1 | 1 | 1 | 1 | 1 | 1 | 1 | 1 | 1 | 1 | 1 | 1 | 1 | 1 | 1 |
| Item-23b | 1 | 1 | 1 | 1 | 1 | 1 | 0 | 1 | 1 | 1 | 0 | 1 | 1 | 1 | 1 | 1 | 1 | 0 | 1 | 1 | 1 | 0 | 1 | 1 |
| Item-23c | 0 | 1 | 0 | 0 | 0 | 1 | 0 | 0 | 0 | 0 | 0 | 0 | 0 | 0 | 0 | 0 | 1 | 0 | 1 | 1 | 0 | 0 | 0 | 0 |
| Item-23d | 1 | 1 | 1 | 1 | 1 | 1 | 1 | 1 | 1 | 1 | 1 | 1 | 1 | 1 | 1 | 1 | 1 | 1 | 1 | 1 | 1 | 1 | 1 | 1 |
| Item-24a | 1 | 1 | 0 | 0 | 0 | 0 | 0 | 0 | 0 | 0 | 0 | 0 | 0 | 0 | 1 | 0 | 0 | 0 | 0 | 0 | 1 | 1 | 0 | 1 |
| Item-24b | 1 | 1 | 0 | 0 | 0 | 0 | 0 | 0 | 0 | 0 | 0 | 0 | 0 | 0 | 1 | 0 | 0 | 0 | 0 | 0 | 1 | 1 | 0 | 1 |
| Item-24c | 0 | 0 | 0 | 0 | 0 | 0 | 0 | 0 | 0 | 0 | 0 | 0 | 0 | 0 | 0 | 0 | 0 | 0 | 0 | 0 | 0 | 0 | 0 | 0 |
| Item-25 | 1 | 1 | 0.5 | 0.5 | 1 | 0.5 | 0 | 0.5 | 1 | 1 | 1 | 1 | 1 | 0.5 | 1 | 0.5 | 0.5 | 1 | 1 | 1 | 1 | 0.5 | 0 | 1 |
| Item-26 | 1 | 1 | 1 | 1 | 1 | 1 | 1 | 1 | 1 | 1 | 1 | 1 | 1 | 1 | 1 | 1 | 1 | 1 | 1 | 1 | 1 | 1 | 1 | 1 |
| Item-27 | 1 | 1 | 1 | 0.5 | 1 | 1 | 0.5 | 1 | 0.5 | 1 | 1 | 0.5 | 1 | 0.5 | 0.5 | 1 | 0.5 | 0.5 | 0.5 | 0.5 | 1 | 1 | 0.5 | 0.5 |
| PRISMA Score | 35 | 36 | 26 | 23.5 | 31 | 25 | 24 | 30 | 26 | 28 | 29 | 26 | 30.5 | 29 | 31.5 | 30.5 | 25.5 | 24 | 28 | 29.5 | 25.5 | 26 | 26.5 | 27.5 |

# Supplementary table S5 Clinical Outcomes in Patients with Breast Cancer and GRADE Scale

## Early breast cancer

| AE Categories | AE | Author(s),year | Trials(subjects) | Population | Experimental intervention | Control intervention | Relative effect | 95%CI | P | I^2^(%) | Risk of bias | Inconsistency | Indirectness | accuracy | publication bias considerations | Overall certainty of evidence |
| --- | --- | --- | --- | --- | --- | --- | --- | --- | --- | --- | --- | --- | --- | --- | --- | --- |
| AE | AEs (all grade) | Agostinetto et al.2021 | 3（12578） | HR+/HER2- early breast cancer | CDK4/6is+ET | ET | OR=9.36 | 3.46-25.33 | <0.001 | 87.5 | -1 | -2 | 0 | 0 | -1 | ⨁◯◯◯  VERY LOW |
|  | AEs (grade≥3) | Agostinetto et al.2021 | 3（12578） | HR+/HER2- early breast cancer | CDK4/6is+ET | ET | OR=11.06 | 5.38-22.74 | <0.001 | 98.3 | -1 | -2 | 0 | 0 | -1 | ⨁◯◯◯  VERY LOW |
|  | G3/4 AEs | Gao et al.2021 | 3（12647） | HR+/HER2- early breast cancer | CDK4/6is+ET | ET | RR=4.14 | 3.33-5.15 | <0.00001 | 91 | -1 | -2 | 0 | 0 | -1 | ⨁◯◯◯  VERY LOW |
| Hematologic AEs | G3/4 Hematologic AEs | Gao et al.2021 | 3（12647） | HR+/HER2- early breast cancer | CDK4/6is+ET | ET | RR=45.96 | 13.57-155.7 | <0.00001 | 99 | -1 | -2 | 0 | -1 | -1 | ⨁◯◯◯  VERY LOW |
|  | G3/4 Neutropenia | Gao et al.2021 | 3（12647） | HR+/HER2- early breast cancer | CDK4/6is+ET | ET | RR=67.39 | 19.56-232.17 | <0.00001 | 92 | -1 | -2 | 0 | 0 | -1 | ⨁◯◯◯  VERY LOW |
|  | G3/4 Leucopenia | Gao et al.2021 | 3（12647） | HR+/HER2- early breast cancer | CDK4/6is+ET | ET | RR=86.69 | 20.52-366.23 | <0.00001 | 87 | -1 | -2 | 0 | 0 | -1 | ⨁◯◯◯  VERY LOW |
|  | G3/4 Anemia | Gao et al.2021 | 3（12647） | HR+/HER2- early breast cancer | CDK4/6is+ET | ET | RR=4.17 | 2.37-7.32 | <0.00001 | 0 | -1 | 0 | 0 | 0 | -1 | ⨁⨁◯◯  LOW |
|  | G3/4 Lymphopenia | Gao et al.2021 | 3（12647） | HR+/HER2- early breast cancer | CDK4/6is+ET | ET | RR=11.12 | 7.20-17.18 | <0.00001 | 0 | -1 | 0 | 0 | 0 | -1 | ⨁⨁◯◯  LOW |
|  | G3/4 Lhrombocytopenia | Gao et al.2021 | 3（12647） | HR+/HER2- early breast cancer | CDK4/6is+ET | ET | RR=8.26 | 2.33-29.34 | 0.001 | 49 | -1 | 0 | 0 | 0 | -1 | ⨁⨁◯◯  LOW |
| Gastrointestinal AEs | G3/4 Nausea | Gao et al.2021 | 3（12647） | HR+/HER2- early breast cancer | CDK4/6is+ET | ET | RR=2.73 | 0.70-10.68 | 0.15 | 49 | -1 | 0 | 0 | 0 | -1 | ⨁⨁◯◯  LOW |
|  | G3/4 Diarrhea | Gao et al.2021 | 3（12647） | HR+/HER2- early breast cancer | CDK4/6is+ET | ET | RR=5.24 | 0.30-92.27 | 0.26 | 93 | -1 | -2 | 0 | 0 | -1 | ⨁◯◯◯  VERY LOW |
| Pain-related adverse effects | G3/4 Fatigue | Gao et al.2021 | 3（12647） | HR+/HER2- early breast cancer | CDK4/6is+ET | ET | RR=5.86 | 1.64-20.93 | 0.006 | 86 | -1 | -2 | 0 | 0 | -1 | ⨁◯◯◯  VERY LOW |
| Other AEs | G3/4 Arthralgia | Gao et al.2021 | 3（12647） | HR+/HER2- early breast cancer | CDK4/6is+ET | ET | RR=0.62 | 0.31-1.25 | 0.18 | 56 | -1 | -1 | 0 | 0 | -1 | ⨁◯◯◯  VERY LOW |

## Advance breast cancer

| AE | Author(s),year | Trials(subjects) | Population | Experimental intervention | Control intervention | Relative effect | 95%CI | P | I^2^(%) | Risk of bias | Inconsistency | Indirectness | accuracy | publication bias considerations | Overall certainty of evidence |
| --- | --- | --- | --- | --- | --- | --- | --- | --- | --- | --- | --- | --- | --- | --- | --- |
| AEs (all grade) | Deng et al.2018 | 6（3186） | HR+/HER2- advanced breast cancer | CDK4/6is+ET | ET/placebo | RR=1.07 | 1.03-1.11 | <0.0002 | 78 | 0 | -1 | 0 | 0 | 0 | ⨁⨁⨁◯  MODERATE |
|  | Ramos-Esquivel et al.2020 | 3（1916） | HR+/HER2- metastatic breast cancer | CDK4/6is+fulvestrant | fulvestrant | OR=1.51 | 0.74-3.08 | 0.26 | 86 | 0 | -1 | 0 | -1 | 0 | ⨁⨁◯◯  LOW |
|  | Martel et al.2017 | 5（2671） | HR+ metastatic breast cancer | CDK4/6is+ET | ET | OR=1.07 | 1.02-1.13 | —— | 85.3 | -1 | -2 | 0 | -1 | -1 | ⨁◯◯◯  VERY LOW |
| G3/4 AEs | Tian et al.2021 | 7（1660） | HR+/HER2- advanced breast cancer | CDK4/6is+ET | ET | RR=2.69 | 2.43,2.97 | <0.00001 | 23 | -1 | 0 | 0 | 0 | 0 | ⨁⨁⨁◯  MODERATE |
|  | Deng et al.2018 | 6（3186） | HR+/HER2- advanced breast cancer | CDK4/6is+ET | ET/placebo | RR=2.81 | 2.54-3.11 | <0.001 | 17.7 | 0 | 0 | 0 | -1 | 0 | ⨁⨁⨁◯  MODERATE |
|  | Zheng et al.2020 | 7（3835） | HR+/HER2- advanced breast cancer | CDK4/6is+ET | ET | RR=2.66 | 2.44-2.90 | <0.00001 | 6 | -1 | 0 | 0 | 0 | -1 | ⨁⨁◯◯  LOW |
|  | Ramos-Esquivel et al.2018 | 3（1827） | post‑menopausal HR+/HER2- metastatic breast cancer | CDK4/6is+AI | AI | OR=7.51 | 6.01-9.38 | <0.00001 | 76 | 0 | -1 | 0 | 0 | 0 | ⨁⨁◯◯  LOW |
|  | Martel et al.2017 | 5（2671） | HR+ metastatic breast cancer | CDK4/6is+ET | ET | OR=2.86 | 2.49-3.27 | —— | 31.6 | -1 | 0 | 0 | 0 | -1 | ⨁⨁◯◯  LOW |
|  | Messina et al.2018 | 8（4578） | HR+/HER2- advanced breast cancer | CDK4/6is+ET | ET | OR=9.64 | 6.00-15.49 | <0.00001 | 90 | -1 | -2 | 0 | 0 | -1 | ⨁◯◯◯  VERY LOW |
| AEs (grade≥3) | Shimoi et al.2020 | 4（1992） | post‑menopausal HR+/HER2- metastatic breast cancer | CDK4/6is+AI | AI | RD=0.43 | 0.39-0.47 | <0.00001 | 75 | -1 | -1 | 0 | 0 | 0 | ⨁⨁◯◯  LOW |
| G3-5 AEs | Li et al.2021 | 6（3689） | HR+/HER2- advanced breast cancer | CDK4/6is+ET | ET | OR=7.32 | 5.54-9.66 | <0.001 | 68.8 | 0 | -1 | 0 | 0 | -1 | ⨁⨁◯◯  LOW |
| Leucopenia | Xu et al.2020 | 8（4580） | HR+/HER2- advanced breast cancer | CDK4/6is+ET | ET | RR=30.65 | 16.51-56.91 | <0.00001 | 0 | -1 | 0 | 0 | 0 | 0 | ⨁⨁⨁◯  MODERATE |
|  | Li et al.2020 | 8（4555） | HR+/HER2- advanced breast cancer | CDK4/6is+ET | ET | RR=10.24 | 7.26-14.44 | <0.00001 | 34 | -1 | 0 | 0 | 0 | 0 | ⨁⨁⨁◯  MODERATE |
|  | Zheng et al.2020 | 8（4555） | HR+/HER2- advanced breast cancer | CDK4/6is+ET | ET | RR=23.82 | 11.10-51.15 | <0.00001 | 30 | -1 | 0 | 0 | 0 | -1 | ⨁⨁◯◯  LOW |
|  | Yang et al.2021 | 6（3685） | HR+/HER2- advanced breast cancer | CDK4/6is+ET | ET | RR=29.33 | 14.8-58.14 | <0.00001 | 0 | 0 | 0 | 0 | -1 | -1 | ⨁⨁◯◯  LOW |
|  | Lin et al.2020 | 6（3401） | HR+/HER2- metastatic breast cancer | CDK4/6is+ET | ET | RR=9.95 | 7.43-13.32 | <0.001 | 46 | -1 | 0 | 0 | 0 | -1 | ⨁⨁◯◯  LOW |
|  | Kassem et al.2018 | 6（3187） | HR+ advanced breast cancer | CDK4/6is+ET | ET | RR=11.31 | 8.06-15.87 | <0.0001 | 0 | -1 | 0 | 0 | 0 | -1 | ⨁⨁◯◯  LOW |
|  | Guo et al.2019 | 3（1352） | cancer/HR+/HER2- advanced breast cancer | palbociclib+ET | ET | OR=25.502 | 14.707-44.220 | 0 | —— | -1 | -2 | 0 | 0 | -1 | ⨁◯◯◯  VERY LOW |
| G3/4 Leucopenia | Li et al.2020 | 8（4555） | HR+/HER2- advanced breast cancer | CDK4/6is+ET | ET | RR=22.08 | 12.12-40.21 | <0.00001 | 0 | -1 | 0 | 0 | 0 | 0 | ⨁⨁⨁◯  MODERATE |
|  | Tian et al.2021 | 8（4555） | HR+/HER2- advanced breast cancer | CDK4/6is+ET | ET | RR=20.96 | 11.81-37.22 | <0.00001 | 0 | -1 | 0 | 0 | 0 | 0 | ⨁⨁⨁◯  MODERATE |
|  | Li et al.2020 | 5（4508） | HR+/HER2- metastatic breast cancer | CDK4/6is+ET | ET | OR=36.36 | 19.35-68.34 | <0.00001 | 0 | -1 | 0 | 0 | 0 | -1 | ⨁⨁◯◯  LOW |
|  | Lin et al.2020 | 6（3401） | HR+/HER2- metastatic breast cancer | CDK4/6is+ET | ET | RR=25.58 | 13.23-49.46 | <0.001 | 0 | -1 | 0 | 0 | 0 | -1 | ⨁⨁◯◯  LOW |
|  | Kassem et al.2018 | 6（3187） | HR+ advanced breast cancer | CDK4/6is+ET | ET | RR=33.86 | 14.59-78.57 | <0.0001 | 0 | -1 | 0 | 0 | 0 | -1 | ⨁⨁◯◯  LOW |
|  | Toss et al.2019 | 4（2499） | HR+ Bone-Only Metastatic Breast Cancer | CDK4/6is+ET | ET | OR=10.405 | 7.458-14.515 | 0 | 48.723 | -1 | 0 | 0 | 0 | -1 | ⨁⨁◯◯  LOW |
|  | Martel et al.2017 | 5（2671） | HR+ metastatic breast cancer | CDK4/6is+ET | ET | OR=33.58 | 14.50-77.77 | —— | 0 | -1 | 0 | 0 | 0 | -1 | ⨁⨁◯◯  LOW |
|  | Guo et al.2019 | 3（1352） | cancer/HR+/HER2- advanced breast cancer | palbociclib+ET | ET | OR=42.988 | 13.589-135.989 | 0 | —— | -1 | -2 | 0 | 0 | -1 | ⨁◯◯◯  VERY LOW |
| Neutropenia | Li et al.2020 | 8（4555） | HR+/HER2- advanced breast cancer | CDK4/6is+ET | ET | RR=14.24 | 10.91-18.59 | <0.00001 | 30 | -1 | 0 | 0 | 0 | 0 | ⨁⨁⨁◯  MODERATE |
|  | Xu et al.2020 | 8（4580） | HR+/HER2- advanced breast cancer | CDK4/6is+ET | ET | RR=32.04 | 17.14-59.90 | <0.00001 | 52 | -1 | -1 | 0 | 0 | 0 | ⨁⨁◯◯  LOW |
|  | Yang et al.2021 | 6（3685） | HR+/HER2- advanced breast cancer | CDK4/6is+ET | ET | RR=28.86 | 15.01-55.48 | <0.00001 | 55 | 0 | -1 | 0 | 0 | -1 | ⨁⨁◯◯  LOW |
|  | Kassem et al.2018 | 6（3187） | HR+ advanced breast cancer | CDK4/6is+ET | ET | RR=14.86 | 11.37-19.41 | <0.0001 | 0 | -1 | 0 | 0 | 0 | -1 | ⨁⨁◯◯  LOW |
|  | Zheng et al.2020 | 8（4555） | HR+/HER2- advanced breast cancer | CDK4/6is+ET | ET | RR=33.57 | 16.23-69.43 | <0.00001 | 64 | -1 | -1 | 0 | 0 | -1 | ⨁◯◯◯  VERY LOW |
|  | Lin et al.2020 | 6（3401） | HR+/HER2- metastatic breast cancer | CDK4/6is+ET | ET | RR=14.77 | 10.26-21.26 | <0.001 | 50.4 | -1 | -1 | 0 | 0 | -1 | ⨁◯◯◯  VERY LOW |
|  | Guo et al.2019 | 3（1352） | cancer/HR+/HER2- advanced breast cancer | palbociclib+ET | ET | OR=72.277 | 44.499-117.396 | 0 | —— | -1 | -2 | 0 | 0 | -1 | ⨁◯◯◯  VERY LOW |
|  | Lee et al.2019 | 2（1334） | HR+/HER2- advanced breast cancer | CDK4/6is+ET | ET | RR=13.15 | 9.34-18.51 | <0.00001 | 57 | -1 | -1 | 0 | 0 | -1 | ⨁◯◯◯  VERY LOW |
| G3/4 Neutropenia | Li et al.2020 | 8（4555） | HR+/HER2- advanced breast cancer | CDK4/6is+ET | ET | RR=31.95 | 17.75-57.50 | <0.00001 | 44 | -1 | 0 | 0 | 0 | 0 | ⨁⨁⨁◯  MODERATE |
|  | Tian et al.2021 | 8（4555） | HR+/HER2- advanced breast cancer | CDK4/6is+ET | ET | RR=32.40 | 17.42-60.25 | <0.00001 | 52 | -1 | -1 | 0 | 0 | 0 | ⨁⨁◯◯  LOW |
|  | Li et al.2020 | 5（4508） | HR+/HER2- metastatic breast cancer | CDK4/6is+ET | ET | OR=57.05 | 38.26-85.05 | <0.00001 | 46 | -1 | 0 | 0 | 0 | -1 | ⨁⨁◯◯  LOW |
|  | Kassem et al.2018 | 6（3187） | HR+ advanced breast cancer | CDK4/6is+ET | ET | RR=44 | 24.72-78.33 | <0.0001 | 0 | -1 | 0 | 0 | 0 | -1 | ⨁⨁◯◯  LOW |
|  | Toss et al.2019 | 4（2499） | HR+ Bone-Only Metastatic Breast Cancer | CDK4/6is+ET | ET | OR=46.755 | 34.903-62.632 | 0 | 0 | -1 | 0 | 0 | 0 | -1 | ⨁⨁◯◯  LOW |
|  | Martel et al.2017 | 5（2671） | HR+ metastatic breast cancer | CDK4/6is+ET | ET | OR=40.77 | 19.52-85.19 | —— | 35.8 | -1 | 0 | 0 | 0 | -1 | ⨁⨁◯◯  LOW |
|  | Messina et al.2018 | 8（4578） | HR+/HER2- advanced breast cancer | CDK4/6is+ET | ET | OR=10.88 | 6.53-18.14 | <0.00001 | 88 | -1 | -2 | 0 | 0 | -1 | ⨁◯◯◯  VERY LOW |
|  | Lin et al.2020 | 6（3401） | HR+/HER2- metastatic breast cancer | CDK4/6is+ET | ET | RR=37.15 | 15.33-90.04 | <0.001 | 65.1 | -1 | -1 | 0 | 0 | -1 | ⨁◯◯◯  VERY LOW |
|  | Guo et al.2019 | 3（1352） | cancer/HR+/HER2- advanced breast cancer | palbociclib+ET | ET | OR=154.215 | 63.023-377.360 | 0 | —— | -1 | -2 | 0 | 0 | -1 | ⨁◯◯◯  VERY LOW |
| Febrile Neutropenia | Yang et al.2021 | 6（3685） | HR+/HER2- advanced breast cancer | CDK4/6is+ET | ET | RR=4.31 | 1.33-13.99 | 0.01 | 0 | 0 | 0 | 0 | -1 | -1 | ⨁⨁◯◯  LOW |
| G3/4 Febrile Neutropenia | Kassem et al.2018 | 6（3187） | HR+ advanced breast cancer | CDK4/6is+ET | ET | RR=3.29 | 0.93-11.57 | 0.06 | 0 | -1 | 0 | 0 | -1 | -1 | ⨁◯◯◯  VERY LOW |
|  | Martel et al.2017 | 5（2671） | HR+ metastatic breast cancer | CDK4/6is+ET | ET | OR=3.99 | 1.09-14.64 | —— | 0 | -1 | 0 | 0 | 0 | -1 | ⨁⨁◯◯  LOW |
| Thrombocytopenia | Yang et al.2021 | 6（3685） | HR+/HER2- advanced breast cancer | CDK4/6is+ET | ET | RR=2.84 | 1.47-5.47 | 0.002 | 0 | 0 | 0 | 0 | -1 | -1 | ⨁⨁◯◯  LOW |
|  | Kassem et al.2018 | 6（3187） | HR+ advanced breast cancer | CDK4/6is+ET | ET | RR=9.04 | 3.78-21.63 | <0.0001 | 70 | -1 | -1 | 0 | 0 | -1 | ⨁◯◯◯  VERY LOW |
|  | Guo et al.2019 | 3（1352） | cancer/HR+/HER2- advanced breast cancer | palbociclib+ET | ET | OR=17.359 | 6.686-45.065 | 0 | —— | -1 | -2 | 0 | 0 | -1 | ⨁◯◯◯  VERY LOW |
| G3/4 Thrombocytopenia | Kassem et al.2018 | 6（3187） | HR+ advanced breast cancer | CDK4/6is+ET | ET | RR=5.7 | 2.03-16.01 | 0.001 | 0 | -1 | 0 | 0 | 0 | -1 | ⨁⨁◯◯  LOW |
|  | Toss et al.2019 | 2（1338） | HR+ Bone-Only Metastatic Breast Cancer | CDK4/6is+ET | ET | OR=7.038 | 2.149-23.052 | 0.001 | 58.296 | -1 | -1 | 0 | 0 | 0 | ⨁⨁◯◯  LOW |
|  | Martel et al.2017 | 5（2671） | HR+ metastatic breast cancer | CDK4/6is+ET | ET | OR=6.02 | 1.81-20.03 | —— | 0 | -1 | 0 | 0 | 0 | -1 | ⨁⨁◯◯  LOW |
|  | Guo et al.2019 | 3（1352） | cancer/HR+/HER2- advanced breast cancer | palbociclib+ET | ET | OR=6.909 | 1.279-37.329 | 0.025 | —— | -1 | -2 | 0 | 0 | -1 | ⨁◯◯◯  VERY LOW |
| Anemia | Xu et al.2020 | 8（4580） | HR+/HER2- advanced breast cancer | CDK4/6is+ET | ET | RR=2.82 | 1.85-4.29 | <0.00001 | 0 | -1 | 0 | 0 | 0 | 0 | ⨁⨁⨁◯  MODERATE |
|  | Li et al.2020 | 8（4555） | HR+/HER2- advanced breast cancer | CDK4/6is+ET | ET | RR=3.52 | 2.57-4.83 | <0.00001 | 62 | -1 | -1 | 0 | 0 | 0 | ⨁⨁◯◯  LOW |
|  | Zheng et al.2020 | 8（4555） | HR+/HER2- advanced breast cancer | CDK4/6is+ET | ET | RR=2.51 | 1.64-3.83 | <0.0001 | 9 | -1 | 0 | 0 | 0 | -1 | ⨁⨁◯◯  LOW |
|  | Yang et al.2021 | 6（3685） | HR+/HER2- advanced breast cancer | CDK4/6is+ET | ET | RR=2.58 | 1.56-4.26 | 0.0002 | 26 | 0 | 0 | 0 | -1 | -1 | ⨁⨁◯◯  LOW |
|  | Lin et al.2020 | 6（3401） | HR+/HER2- metastatic breast cancer | CDK4/6is+ET | ET | RR=3.53 | 2.36-5.26 | <0.001 | 67.9 | -1 | -1 | 0 | 0 | -1 | ⨁◯◯◯  VERY LOW |
|  | Kassem et al.2018 | 6（3187） | HR+ advanced breast cancer | CDK4/6is+ET | ET | RR=3.57 | 2.65-4.81 | <0.0001 | 53 | -1 | -1 | 0 | 0 | -1 | ⨁◯◯◯  VERY LOW |
|  | Guo et al.2019 | 3（1352） | cancer/HR+/HER2- advanced breast cancer | palbociclib+ET | ET | OR=3.504 | 2.481-4.950 | 0 | —— | -1 | -2 | 0 | 0 | -1 | ⨁◯◯◯  VERY LOW |
| G3/4 Anemia | Tian et al.2021 | 8（4555） | HR+/HER2- advanced breast cancer | CDK4/6is+ET | ET | RR=2.42 | 1.55-3.77 | 0.0001 | 0 | -1 | 0 | 0 | 0 | 0 | ⨁⨁⨁◯  MODERATE |
|  | Li et al.2020 | 8（4555） | HR+/HER2- advanced breast cancer | CDK4/6is+ET | ET | RR=2.24 | 1.41-3.56 | 0.0007 | 11 | -1 | 0 | 0 | 0 | 0 | ⨁⨁⨁◯  MODERATE |
|  | Lin et al.2020 | 6（3401） | HR+/HER2- metastatic breast cancer | CDK4/6is+ET | ET | RR=2.24 | 1.38-3.85 | 0.001 | 24.6 | -1 | 0 | 0 | 0 | -1 | ⨁⨁◯◯  LOW |
|  | Kassem et al.2018 | 6（3187） | HR+ advanced breast cancer | CDK4/6is+ET | ET | RR=2.8 | 1.45-5.41 | 0.002 | 16 | -1 | 0 | 0 | 0 | -1 | ⨁⨁◯◯  LOW |
|  | Martel et al.2017 | 5（2671） | HR+ metastatic breast cancer | CDK4/6is+ET | ET | OR=2.66 | 1.30-5.45 | —— | 26.8 | -1 | 0 | 0 | 0 | -1 | ⨁⨁◯◯  LOW |
|  | Toss et al.2019 | 4（2499） | HR+ Bone-Only Metastatic Breast Cancer | CDK4/6is+ET | ET | OR=3.745 | 2.40-5.845 | 0 | 62.709 | -1 | -1 | 0 | 0 | -1 | ⨁◯◯◯  VERY LOW |
|  | Guo et al.2019 | 3（1352） | cancer/HR+/HER2- advanced breast cancer | palbociclib+ET | ET | OR=2.654 | 1.225-5.750 | 0.013 | —— | -1 | -2 | 0 | 0 | -1 | ⨁◯◯◯  VERY LOW |
| Diarrhea | Xu et al.2020 | 8（4580） | HR+/HER2- advanced breast cancer | CDK4/6is+ET | ET | RR=2.85 | 1.10-7.42 | 0.03 | 58 | -1 | -1 | 0 | 0 | 0 | ⨁⨁◯◯  LOW |
|  | Shohdy et al.2017 | 4（2016） | HR+ advanced breast cancer | CDK4/6is+ET | ET | RR=1.44 | 1.19-1.74 | 0.0002 | 13 | -1 | 0 | 0 | 0 | -1 | ⨁⨁◯◯  LOW |
|  | Li et al.2020 | 8（4555） | HR+/HER2- advanced breast cancer | CDK4/6is+ET | ET | RR=1.71 | 1.23-2.37 | 0.001 | 89 | -1 | -2 | 0 | 0 | 0 | ⨁◯◯◯  VERY LOW |
|  | Lin et al.2020 | 6（3401） | HR+/HER2- metastatic breast cancer | CDK4/6is+ET | ET | RR=1.64 | 1.08-2.48 | 0.02 | 90.3 | -1 | -2 | 0 | -1 | -1 | ⨁◯◯◯  VERY LOW |
|  | Yang et al.2021 | 6（3685） | HR+/HER2- advanced breast cancer | CDK4/6is+ET | ET | RR=3.99 | 1.05-15.10 | 0.04 | 70 | 0 | -1 | 0 | -1 | -1 | ⨁◯◯◯  VERY LOW |
| G3/4 Diarrhea | Tian et al.2021 | 8（4555） | HR+/HER2- advanced breast cancer | CDK4/6is+ET | ET | RR=2.88 | 1.01-8.22 | 0.05 | 61 | -1 | 0 | 0 | 0 | 0 | ⨁⨁⨁◯  MODERATE |
|  | Li et al.2020 | 8（4555） | HR+/HER2- advanced breast cancer | CDK4/6is+ET | ET | RR=2.60 | 0.94-7.16 | 0.06 | 57 | -1 | -1 | 0 | 0 | -1 | ⨁◯◯◯  VERY LOW |
|  | Li et al.2020 | 5（4508） | HR+/HER2- metastatic breast cancer | CDK4/6is+ET | ET | OR=4.97 | 2.84-8.69 | <0.00001 | 62 | -1 | -1 | 0 | 0 | -1 | ⨁◯◯◯  VERY LOW |
|  | Lin et al.2020 | 6（3401） | HR+/HER2- metastatic breast cancer | CDK4/6is+ET | ET | RR=2.51 | 0.55-11.42 | 0.235 | 67.2 | -1 | -1 | 0 | -1 | -1 | ⨁◯◯◯  VERY LOW |
|  | Martel et al.2017 | 5（2671） | HR+ metastatic breast cancer | CDK4/6is+ET | ET | OR=2.26 | 0.39-13.10 | —— | 73.9 | -1 | -1 | 0 | 0 | -1 | ⨁◯◯◯  VERY LOW |
|  | Toss et al.2019 | 4（2499） | HR+ Bone-Only Metastatic Breast Cancer | CDK4/6is+ET | ET | OR=2.365 | 0.969-5,77 | 0.059 | 95.359 | -1 | -2 | 0 | -1 | -1 | ⨁◯◯◯  VERY LOW |
|  | Shohdy et al.2017 | 4（2016） | HR+ advanced breast cancer | CDK4/6is+ET | ET | RR=1.19 | 0.44-3.21 | 0.73 | 0 | -1 | 0 | 0 | -1 | -1 | ⨁◯◯◯  VERY LOW |
| Nausea | Li et al.2020 | 8（4555） | HR+/HER2- advanced breast cancer | CDK4/6is+ET | ET | RR=1.63 | 1.44-1.84 | <0.00001 | 37 | -1 | 0 | 0 | 0 | 0 | ⨁⨁⨁◯  MODERATE |
|  | Xu et al.2020 | 8（4580） | HR+/HER2- advanced breast cancer | CDK4/6is+ET | ET | RR=1.51 | 0.85-2.68 | 0.16 | 32 | -1 | 0 | 0 | -1 | 0 | ⨁⨁◯◯  LOW |
|  | Yang et al.2021 | 6（3685） | HR+/HER2- advanced breast cancer | CDK4/6is+ET | ET | RR=3.18 | 1.20-8.42 | 0.02 | 0 | 0 | 0 | 0 | -1 | -1 | ⨁⨁◯◯  LOW |
|  | Lin et al.2020 | 6（3401） | HR+/HER2- metastatic breast cancer | CDK4/6is+ET | ET | RR=1.66 | 1.49-1.85 | <0.001 | 36.8 | -1 | 0 | 0 | 0 | -1 | ⨁⨁◯◯  LOW |
|  | Shohdy et al.2017 | 4（2016） | HR+ advanced breast cancer | CDK4/6is+ET | ET | RR=1.48 | 1.12-1.93 | 0.005 | 68 | -1 | -1 | 0 | 0 | -1 | ⨁◯◯◯  VERY LOW |
| G3/4 Nausea | Li et al.2020 | 8（4555） | HR+/HER2- advanced breast cancer | CDK4/6is+ET | ET | RR=1.33 | 0.59-2.99 | 0.5 | 29 | -1 | 0 | 0 | -1 | 0 | ⨁⨁◯◯  LOW |
|  | Lin et al.2020 | 6（3401） | HR+/HER2- metastatic breast cancer | CDK4/6is+ET | ET | RR=2.19 | 1.07-4.49 | 0.032 | 0 | -1 | 0 | 0 | 0 | -1 | ⨁⨁◯◯  LOW |
|  | Martel et al.2017 | 5（2671） | HR+ metastatic breast cancer | CDK4/6is+ET | ET | OR=1.13 | 0.29-4.48 | —— | 56.5 | -1 | 0 | 0 | 0 | -1 | ⨁⨁◯◯  LOW |
|  | Toss et al.2019 | 4（2499） | HR+ Bone-Only Metastatic Breast Cancer | CDK4/6is+ET | ET | OR=2.081 | 1.738-2.493 | 0 | 46.511 | -1 | 0 | 0 | 0 | -1 | ⨁⨁◯◯  LOW |
|  | Li et al.2020 | 5（4508） | HR+/HER2- metastatic breast cancer | CDK4/6is+ET | ET | OR=1.47 | 0.81-2.66 | 0.2 | 27 | -1 | 0 | 0 | -1 | -1 | ⨁◯◯◯  VERY LOW |
|  | Shohdy et al.2017 | 4（2016） | HR+ advanced breast cancer | CDK4/6is+ET | ET | RR=1.10 | 0.29-4.13 | 0.89 | 46 | -1 | 0 | 0 | -1 | -1 | ⨁◯◯◯  VERY LOW |
| Vomiting | Xu et al.2020 | 7（3983） | HR+/HER2- advanced breast cancer | CDK4/6is+ET | ET | RR=1.31 | 0.74- 2.32 | 0.36 | 46 | -1 | 0 | 0 | -1 | 0 | ⨁⨁◯◯  LOW |
|  | Yang et al.2021 | 6（3685） | HR+/HER2- advanced breast cancer | CDK4/6is+ET | ET | RR=1.08 | 0.41-2.86 | 0.15 | 53 | 0 | -1 | 0 | -1 | -1 | ⨁◯◯◯  VERY LOW |
|  | Lin et al.2020 | 6（3401） | HR+/HER2- metastatic breast cancer | CDK4/6is+ET | ET | RR=1.74 | 1.29-2.34 | <0.001 | 67.3 | -1 | -1 | 0 | -1 | -1 | ⨁◯◯◯  VERY LOW |
|  | Shohdy et al.2017 | 3（1350） | HR+ advanced breast cancer | CDK4/6is+ET | ET | RR=1.74 | 1.09-2.76 | 0.02 | 55 | -1 | -1 | 0 | 0 | -1 | ⨁◯◯◯  VERY LOW |
| G3/4 Vomiting | Li et al.2020 | 5（4508） | HR+/HER2- metastatic breast cancer | CDK4/6is+ET | ET | OR=1.32 | 0.76-2.29 | 0.32 | 40 | -1 | 0 | 0 | -1 | -1 | ⨁◯◯◯  VERY LOW |
|  | Lin et al.2020 | 6（3401） | HR+/HER2- metastatic breast cancer | CDK4/6is+ET | ET | RR=1.75 | 0.92-3.32 | 0.087 | 45.3 | -1 | 0 | 0 | -1 | -1 | ⨁◯◯◯  VERY LOW |
|  | Shohdy et al.2017 | 3（1350） | HR+ advanced breast cancer | CDK4/6is+ET | ET | RR=1.38 | 0.25-7.75 | 0.72 | 42 | -1 | 0 | 0 | -1 | -1 | ⨁◯◯◯  VERY LOW |
|  | Toss et al.2019 | 4（2499） | HR+ Bone-Only Metastatic Breast Cancer | CDK4/6is+ET | ET | OR=1.676 | 0.974-2.884 | 0.062 | 84.463 | -1 | -2 | 0 | -1 | -1 | ⨁◯◯◯  VERY LOW |
| Decreased Appetite | Yang et al.2021 | 6（3685） | HR+/HER2- advanced breast cancer | CDK4/6is+ET | ET | RR=3.83 | 1.01-14.56 | 0.05 | 0 | 0 | 0 | 0 | -1 | -1 | ⨁⨁◯◯  LOW |
|  | Shohdy et al.2017 | 3（1350） | HR+ advanced breast cancer | CDK4/6is+ET | ET | RR=1.42 | 1.07-1.88 | 0.02 | 0 | -1 | 0 | 0 | 0 | -1 | ⨁⨁◯◯  LOW |
| G3/4 Decreased Appetite | Toss et al.2019 | 4（2499） | HR+ Bone-Only Metastatic Breast Cancer | CDK4/6is+ET | ET | OR=1.68 | 1.131-2.496 | 0.01 | 58.753 | -1 | -1 | 0 | 0 | -1 | ⨁◯◯◯  VERY LOW |
|  | Shohdy et al.2017 | 3（1350） | HR+ advanced breast cancer | CDK4/6is+ET | ET | RR=4 | 0.87-18.37 | 0.07 | 0 | -1 | 0 | 0 | -1 | -1 | ⨁◯◯◯  VERY LOW |
|  | Guo et al.2019 | 3（1352） | cancer/HR+/HER2- advanced breast cancer | palbociclib+ET | ET | OR=2.218 | 0.460-10.691 | 0.321 | —— | -1 | -2 | 0 | 0 | -1 | ⨁◯◯◯  VERY LOW |
| G3/4 Constipation | Toss et al.2019 | 4（2499） | HR+ Bone-Only Metastatic Breast Cancer | CDK4/6is+ET | ET | OR=1.370 | 1.106-1.697 | 0.004 | 0 | -1 | 0 | 0 | 0 | 0 | ⨁⨁⨁◯  MODERATE |
| Stomatitis | Lasheen et al.2017 | 2（682） | HR+ advanced breast cancer | CDK4/6is+ET | ET | RR=4.87 | 2.11-11.24 | 0.0002 | 70 | -1 | -1 | 0 | 0 | -1 | ⨁◯◯◯  VERY LOW |
| G3/4 Stomatitis | Toss et al.2019 | 2（1338） | HR+ Bone-Only Metastatic Breast Cancer | CDK4/6is+ET | ET | OR=1.950 | 0.921-4.130 | 0.081 | 70.461 | -1 | -1 | 0 | -1 | 0 | ⨁◯◯◯  VERY LOW |
| Mucositis | Guo et al.2019 | 3（1352） | cancer/HR+/HER2- advanced breast cancer | palbociclib+ET | ET | OR=3.649 | 2.208-6.030 | 0 | —— | -1 | -2 | 0 | 0 | -1 | ⨁◯◯◯  VERY LOW |
| Fatigue | Li et al.2020 | 8（4555） | HR+/HER2- advanced breast cancer | CDK4/6is+ET | ET | RR=1.24 | 1.08-1.41 | 0.002 | 51 | -1 | -1 | 0 | 0 | 0 | ⨁⨁◯◯  LOW |
|  | Yang et al.2021 | 6（3685） | HR+/HER2- advanced breast cancer | CDK4/6is+ET | ET | RR=8.39 | 4.27-16.47 | <0.00001 | 15 | 0 | 0 | 0 | -1 | -1 | ⨁⨁◯◯  LOW |
|  | Lasheen et al.2017 | 4（2007） | HR+ advanced breast cancer | CDK4/6is+ET | ET | RR=1.34 | 1.17-1.54 | <0.0001 | 0 | -1 | 0 | 0 | 0 | -1 | ⨁⨁◯◯  LOW |
|  | Lin et al.2020 | 6（3401） | HR+/HER2- metastatic breast cancer | CDK4/6is+ET | ET | RR=1.22 | 1.02-1.45 | 0.029 | 62.3 | -1 | -1 | 0 | -1 | -1 | ⨁◯◯◯  VERY LOW |
| G3/4 Fatigue | Li et al.2020 | 8（4555） | HR+/HER2- advanced breast cancer | CDK4/6is+ET | ET | RR=3.14 | 1.63-6.07 | 0.0007 | 0 | -1 | 0 | 0 | 0 | 0 | ⨁⨁⨁◯  MODERATE |
|  | Tian et al.2021 | 8（4555） | HR+/HER2- advanced breast cancer | CDK4/6is+ET | ET | RR=3.69 | 1.88-7.26 | 0.0002 | 0 | -1 | 0 | 0 | 0 | 0 | ⨁⨁⨁◯  MODERATE |
|  | Li et al.2020 | 5（4508） | HR+/HER2- metastatic breast cancer | CDK4/6is+ET | ET | OR=3.76 | 1.90-7.45 | 0.0001 | 0 | -1 | 0 | 0 | 0 | -1 | ⨁⨁◯◯  LOW |
|  | Lin et al.2020 | 6（3401） | HR+/HER2- metastatic breast cancer | CDK4/6is+ET | ET | RR=3.54 | 1.70-7.40 | 0.001 | 0 | -1 | 0 | 0 | 0 | -1 | ⨁⨁◯◯  LOW |
|  | Toss et al.2019 | 4（2499） | HR+ Bone-Only Metastatic Breast Cancer | CDK4/6is+ET | ET | OR=1.335 | 1.119-1.59 | 0.001 | 40.24 | -1 | 0 | 0 | 0 | -1 | ⨁⨁◯◯  LOW |
|  | Lasheen et al.2017 | 4（2007） | HR+ advanced breast cancer | CDK4/6is+ET | ET | RR=2.40 | 1.10-5.26 | 0.03 | 0 | -1 | 0 | 0 | 0 | -1 | ⨁⨁◯◯  LOW |
|  | Martel et al.2017 | 5（2671） | HR+ metastatic breast cancer | CDK4/6is+ET | ET | OR=3.24 | 1.51-6.98 | —— | 0 | -1 | 0 | 0 | 0 | -1 | ⨁⨁◯◯  LOW |
|  | Guo et al.2019 | 3（1352） | cancer/HR+/HER2- advanced breast cancer | palbociclib+ET | ET | OR=2.852 | 0.961-8.466 | 0.059 | —— | -1 | -2 | 0 | 0 | -1 | ⨁◯◯◯  VERY LOW |
| Hot Flush | Lin et al.2020 | 6（3401） | HR+/HER2- metastatic breast cancer | CDK4/6is+ET | ET | RR=0.96 | 0.83-1.10 | 0.522 | 0 | -1 | 0 | 0 | -1 | -1 | ⨁◯◯◯  VERY LOW |
| G3/4 Hot Flush | Lin et al.2020 | 6（3401） | HR+/HER2- metastatic breast cancer | CDK4/6is+ET | ET | RR=1.11 | 0.25-4.91 | 0.892 | 4.4 | -1 | 0 | 0 | -1 | -1 | ⨁◯◯◯  VERY LOW |
|  | Toss et al.2019 | 3（2006） | HR+ Bone-Only Metastatic Breast Cancer | CDK4/6is+ET | ET | OR=0.843 | 0.612-1.162 | 0.297 | 61.331 | -1 | -1 | 0 | -1 | 0 | ⨁◯◯◯  VERY LOW |
| G3/4 Pyrexia | Toss et al.2019 | 2（1338） | HR+ Bone-Only Metastatic Breast Cancer | CDK4/6is+ET | ET | OR=1.794 | 1.243-2.589 | 0.002 | 0 | -1 | 0 | 0 | 0 | 0 | ⨁⨁⨁◯  MODERATE |
| G3/4 Asthenia | Toss et al.2019 | 2（1338） | HR+ Bone-Only Metastatic Breast Cancer | CDK4/6is+ET | ET | OR=1.266 | 0.909-1.762 | 0.162 | 14.611 | -1 | 0 | 0 | -1 | 0 | ⨁⨁◯◯  LOW |
|  | Guo et al.2019 | 3（1352） | cancer/HR+/HER2- advanced breast cancer | palbociclib+ET | ET | OR=7.362 | 0.920-58.917 | 0.06 | —— | -1 | -2 | 0 | 0 | -1 | ⨁◯◯◯  VERY LOW |
| Increased ALT | Yang et al.2021 | 6（3685） | HR+/HER2- advanced breast cancer | CDK4/6is+ET | ET | RR=4.14 | 2.46-6.95 | <0.00001 | 0 | 0 | 0 | 0 | -1 | -1 | ⨁⨁◯◯  LOW |
| Increased AST | Yang et al.2021 | 6（3685） | HR+/HER2- advanced breast cancer | CDK4/6is+ET | ET | RR=2.58 | 1.02-6.57 | 0.05 | 57 | 0 | -1 | 0 | -1 | -1 | ⨁◯◯◯  VERY LOW |
| G3/4 Increased ALT | Toss et al.2019 | 3（1833） | HR+ Bone-Only Metastatic Breast Cancer | CDK4/6is+ET | ET | OR=2.697 | 1.582-4.599 | 0 | 56.966 | -1 | -1 | 0 | 0 | 0 | ⨁⨁◯◯  LOW |
|  | Martel et al.2017 | 5（2671） | HR+ metastatic breast cancer | CDK4/6is+ET | ET | OR=4.33 | 2.16-8.72 | —— | 0 | -1 | 0 | 0 | 0 | -1 | ⨁⨁◯◯  LOW |
| G3/4 Increased AST | Toss et al.2019 | 2（1338） | HR+ Bone-Only Metastatic Breast Cancer | CDK4/6is+ET | ET | OR=2.498 | 0.761-8.192 | 0.131 | 88.101 | -1 | -2 | 0 | -1 | 0 | ⨁◯◯◯  VERY LOW |
|  | Martel et al.2017 | 5（2671） | HR+ metastatic breast cancer | CDK4/6is+ET | ET | OR=1.86 | 0.74-4.64 | —— | 46.1 | -1 | 0 | 0 | 0 | -1 | ⨁⨁◯◯  LOW |
| VTE | Thein et al.2020 | 8（4557） | HR+/HER2- metastatic breast cancer | CDK4/6is+ET | ET | RR=2.62 | 1.21-5.65 | P=0.01 | 15 | 0 | 0 | 0 | 0 | 0 | ⨁⨁⨁⨁  HIGH |
|  | Bolzacchini et al.2021 | 7（4226） | HR+/HER2- advanced breast cancer | CDK4/6is+ET | ET | OR=2.90 | 1.32-6.37 | P=0.008 | 0 | -1 | 0 | 0 | 0 | -1 | ⨁⨁◯◯  LOW |
| G1/2 VTE | Bolzacchini et al.2021 | 7（4226） | HR+/HER2- advanced breast cancer | CDK4/6is+ET | ET | OR=4.08 | 0.94-17.84 | —— | 0 | -1 | 0 | 0 | -1 | -1 | ⨁◯◯◯  VERY LOW |
| G3/4 VTE | Bolzacchini et al.2021 | 7（4226） | HR+/HER2- advanced breast cancer | CDK4/6is+ET | ET | OR=1.42 | 0.59-3.44 | —— | 0 | -1 | 0 | 0 | -1 | -1 | ⨁◯◯◯  VERY LOW |
| G5 VTE | Bolzacchini et al.2021 | 7（4226） | HR+/HER2- advanced breast cancer | CDK4/6is+ET | ET | OR=1.85 | 0.30-11.26 | —— | 0 | -1 | 0 | 0 | -1 | -1 | ⨁◯◯◯  VERY LOW |
| All Arterial Events | Bolzacchini et al.2021 | 7（4226） | HR+/HER3- advanced breast cancer | CDK4/6is+ET | ET | OR=1.22 | 0.47-3.18 | —— | 0 | -1 | 0 | 0 | -1 | -1 | ⨁◯◯◯  VERY LOW |
| Myocardial Infarction | Bolzacchini et al.2021 | 7（4226） | HR+/HER4- advanced breast cancer | CDK4/6is+ET | ET | OR=1.08 | 0.30-3.93 | —— | 0 | -1 | 0 | 0 | -1 | -1 | ⨁◯◯◯  VERY LOW |
| Angina | Bolzacchini et al.2021 | 7（4226） | HR+/HER5- advanced breast cancer | CDK4/6is+ET | ET | OR=2.01 | 0.22-18.09 | —— | 0 | -1 | 0 | 0 | -1 | -1 | ⨁◯◯◯  VERY LOW |
| Cerebral Ischemia | Bolzacchini et al.2021 | 7（4226） | HR+/HER6- advanced breast cancer | CDK4/6is+ET | ET | OR=1.51 | 0.16-14.57 | —— | 0 | -1 | 0 | 0 | -1 | -1 | ⨁◯◯◯  VERY LOW |
| Alopecia | Lasheen et al.2017 | 4（2007） | HR+ advanced breast cancer | CDK4/6is+ET | ET | RR=2.14 | 1.23-3.73 | 0.007 | 0 | -1 | 0 | 0 | 0 | -1 | ⨁⨁◯◯  LOW |
|  | Guo et al.2019 | 3（1352） | cancer/HR+/HER2- advanced breast cancer | palbociclib+ET | ET | OR=2.903 | 2.062-4.086 | 0 | —— | -1 | -2 | 0 | 0 | -1 | ⨁◯◯◯  VERY LOW |
| G3/4 Alopecia | Toss et al.2019 | 4（2499） | HR+ Bone-Only Metastatic Breast Cancer | CDK4/6is+ET | ET | OR=2.472 | 1.995-3.063 | 0 | 7.913 | -1 | 0 | 0 | 0 | -1 | ⨁⨁◯◯  LOW |
| Rash | Guo et al.2019 | 3（1352） | cancer/HR+/HER2- advanced breast cancer | palbociclib+ET | ET | OR=2.157 | 1.122-4.149 | 0.021 | —— | -1 | -2 | 0 | 0 | -1 | ⨁◯◯◯  VERY LOW |
| G3/4 Rash | Toss et al.2019 | 3（1833） | HR+ Bone-Only Metastatic Breast Cancer | CDK4/6is+ET | ET | OR=1.992 | 1.335-2.981 | 0.001 | 52.54 | -1 | -1 | 0 | 0 | 0 | ⨁⨁◯◯  LOW |
|  | Martel et al.2017 | 5（2671） | HR+ metastatic breast cancer | CDK4/6is+ET | ET | OR=3.13 | 0.81-12.11 | —— | 0 | -1 | 0 | 0 | 0 | -1 | ⨁⨁◯◯  LOW |
|  | Guo et al.2019 | 3（1352） | cancer/HR+/HER2- advanced breast cancer | palbociclib+ET | ET | OR=2.169 | 0.365-12.875 | 0.394 | —— | -1 | -2 | 0 | 0 | -1 | ⨁◯◯◯  VERY LOW |
| Headache | Lin et al.2020 | 6（3401） | HR+/HER2- metastatic breast cancer | CDK4/6is+ET | ET | RR=1.12 | 0.97-1.28 | 0.111 | 0 | -1 | 0 | 0 | -1 | -1 | ⨁◯◯◯  VERY LOW |
| G3/4 Headache | Toss et al.2019 | 4（2499） | HR+ Bone-Only Metastatic Breast Cancer | CDK4/6is+ET | ET | OR=1.013 | 0.833-1.231 | 0.899 | 44.087 | -1 | 0 | 0 | -1 | 0 | ⨁⨁◯◯  LOW |
|  | Lin et al.2020 | 6（3401） | HR+/HER2- metastatic breast cancer | CDK4/6is+ET | ET | RR=1.00 | 0.36-2.78 | 0.997 | 0 | -1 | 0 | 0 | -1 | -1 | ⨁◯◯◯  VERY LOW |
| Arthralgia | Li et al.2020 | 7（4067） | HR+/HER2- advanced breast cancer | CDK4/6is+ET | ET | RR=0.98 | 0.87-1.09 | 0.67 | 0 | -1 | 0 | 0 | -1 | 0 | ⨁⨁◯◯  LOW |
|  | Lin et al.2020 | 6（3401） | HR+/HER2- metastatic breast cancer | CDK4/6is+ET | ET | RR=0.97 | 0.86-1.10 | 0.649 | 0 | -1 | 0 | 0 | -1 | -1 | ⨁◯◯◯  VERY LOW |
| G3/4 Arthralgia | Toss et al.2019 | 3（2006） | HR+ Bone-Only Metastatic Breast Cancer | CDK4/6is+ET | ET | OR=1.044 | 0.861-1.265 | 0.662 | 0 | -1 | 0 | 0 | -1 | 0 | ⨁⨁◯◯  LOW |
|  | Lin et al.2020 | 6（3401） | HR+/HER2- metastatic breast cancer | CDK4/6is+ET | ET | RR=0.93 | 0.40-2.14 | 0.866 | 0 | -1 | 0 | 0 | -1 | -1 | ⨁◯◯◯  VERY LOW |
| G3/4 Abdominal Pain | Toss et al.2019 | 3（1831） | HR+ Bone-Only Metastatic Breast Cancer | CDK4/6is+ET | ET | OR=2.182 | 1.570-3.032 | 0 | 38.116 | -1 | 0 | 0 | 0 | 0 | ⨁⨁⨁◯  MODERATE |
| G3/4 Pain In Extremities | Toss et al.2019 | 2（1338） | HR+ Bone-Only Metastatic Breast Cancer | CDK4/6is+ET | ET | OR=0.913 | 0.658-1.266 | 0.584 | 0 | -1 | 0 | 0 | -1 | 0 | ⨁⨁◯◯  LOW |
| G3/4 Back Pain | Toss et al.2019 | 3（2006） | HR+ Bone-Only Metastatic Breast Cancer | CDK4/6is+ET | ET | OR=1.055 | 0.846-1.315 | 0.636 | 0 | -1 | 0 | 0 | -1 | 0 | ⨁⨁◯◯  LOW |
| G3/4 Cough | Toss et al.2019 | 3（2006） | HR+ Bone-Only Metastatic Breast Cancer | CDK4/6is+ET | ET | OR=1.272 | 1.009-1.603 | 0.042 | 0 | -1 | 0 | 0 | 0 | 0 | ⨁⨁⨁◯  MODERATE |
